# *S*-Adenosylmethionine: more than just a methyl donor

**DOI:** 10.1039/d2np00086e

**Published:** 2023-03-09

**Authors:** Yu-Hsuan Lee, Daan Ren, Byungsun Jeon, Hung-wen Liu

**Affiliations:** a Department of Chemistry, University of Texas at Austin Austin TX 78712 USA H.w.liu@mail.utexas.edu; b Division of Chemical Biology & Medicinal Chemistry, College of Pharmacy, University of Texas at Austin Austin TX 78712 USA

## Abstract

Covering: from 2000 up to the very early part of 2023

*S*-Adenosyl-l-methionine (SAM) is a naturally occurring trialkyl sulfonium molecule that is typically associated with biological methyltransfer reactions. However, SAM is also known to donate methylene, aminocarboxypropyl, adenosyl and amino moieties during natural product biosynthetic reactions. The reaction scope is further expanded as SAM itself can be modified prior to the group transfer such that a SAM-derived carboxymethyl or aminopropyl moiety can also be transferred. Moreover, the sulfonium cation in SAM has itself been found to be critical for several other enzymatic transformations. Thus, while many SAM-dependent enzymes are characterized by a methyltransferase fold, not all of them are necessarily methyltransferases. Furthermore, other SAM-dependent enzymes do not possess such a structural feature suggesting diversification along different evolutionary lineages. Despite the biological versatility of SAM, it nevertheless parallels the chemistry of sulfonium compounds used in organic synthesis. The question thus becomes how enzymes catalyze distinct transformations *via* subtle differences in their active sites. This review summarizes recent advances in the discovery of novel SAM utilizing enzymes that rely on Lewis acid/base chemistry as opposed to radical mechanisms of catalysis. The examples are categorized based on the presence of a methyltransferase fold and the role played by SAM within the context of known sulfonium chemistry.

## Abstract 2

This review summarizes the chemistry of *S*-adenosylmethionine dependent enzymes that goes beyond methylation.

## Graphical abstract

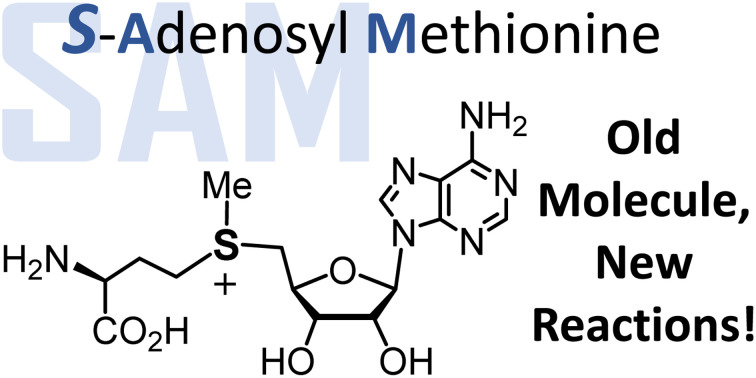

## 1 Introduction

*S*-Adenosyl-l-methionine (SAM or AdoMet, 1) is a sulfonium-containing primary metabolite found in both prokaryotic and eukaryotic cells.^1^ SAM is best known for the role it plays in one-carbon metabolism where it serves as a methyl donor in many biological reactions,^2–4^ and a search in InterPro reveals that approximately 3 million protein sequences have been annotated as SAM-dependent methyltransferases.^5^ The molecular mechanism of SAM-dependent methylation generally involves a nucleophilic substitution at the sulfonium methyl carbon of SAM to generate a variety of *C*-, *O*-, *N*- and *S*-methylated products.^6^

Not all enzymes, however, utilize SAM to catalyze methylation reactions. In particular, a significant number of enzymes also bind an [Fe_4_S_4_] cluster typically *via* a highly conserved CX_3_CX_2_C motif in the active site that serves to reduce SAM leading to the homolytic cleavage of the C5′–S linkage.^7,8^ The resulting 5′-deoxyadenosyl radical equivalent (2) can then initiate a broad range of radical-mediated transformations. Radical SAM enzymes thus constitute a protein superfamily with more than 700 thousand protein sequences^9^ arguably becoming one of the focal points of modern enzymological study as summarized in many recent reviews.^10–17^

A number of other enzymes are also dependent on SAM for their activity; however, they are not easily classified as either SAM-dependent transferases or radical SAM enzymes.^18–21^ Among these enzymes, SAM may serve as an alkyl donor as opposed to a strict methyl donor or stabilize reaction intermediate(s) *via* the positive charge of its cationic sulfonium moiety. Alternatively, SAM may only play a structural role helping to maintain the integrity of the enzyme and its active site and thus remain intact throughout the catalytic cycle. While many of these enzymes are structurally related to the SAM-dependent methyltransferases,^22–24^ this is not universally the case.

**Fig. 1:**
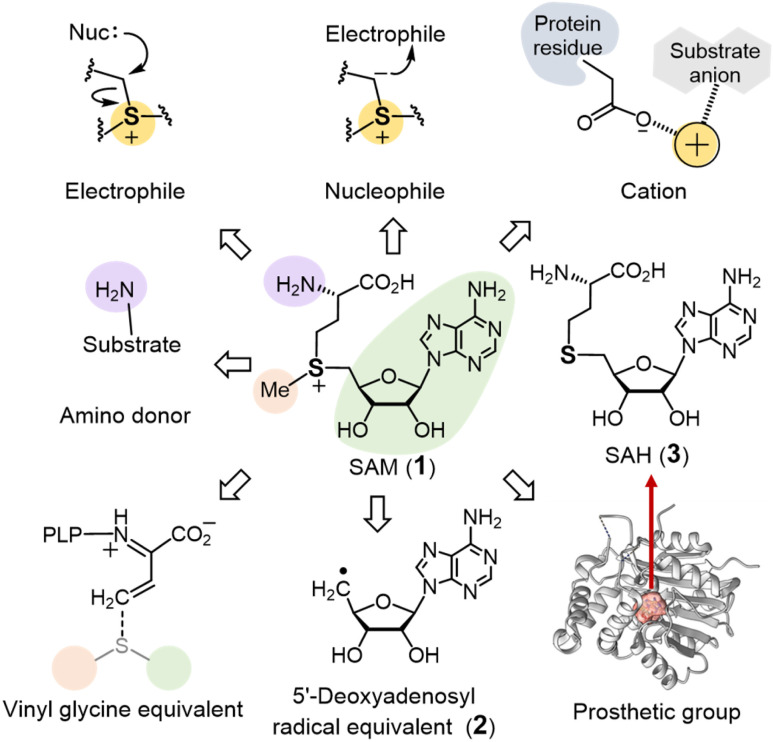
Distinct uses of SAM in enzymatic reactions.

This review emphasizes the varied enzymology of SAM in biological transformations that are not expected to involve radical intermediates (Fig. 1). The enzymes will therefore be discussed based on whether they are structurally related to the SAM-dependent methyltransferases (Section 2) or appear to represent different classes of enzymes altogether (Section 3). As will become evident, however, even structurally related enzymes can exhibit unexpected behavior thereby complicating bioinformatics efforts to infer their activities and placing an emphasis on experimental investigation. Research has nevertheless made steady progress, and since 2010 there has been a considerable expansion in the recognized scope of SAM enzymology observed in nature. Furthermore, continued study in the future is certain to fill in many of the gaps in the current understanding of these enzymes as well as identify new and potentially unforeseen biological reactions involving SAM.

## 2 Enzymes with a methyltransferase (MT) fold

**Fig. 2:**
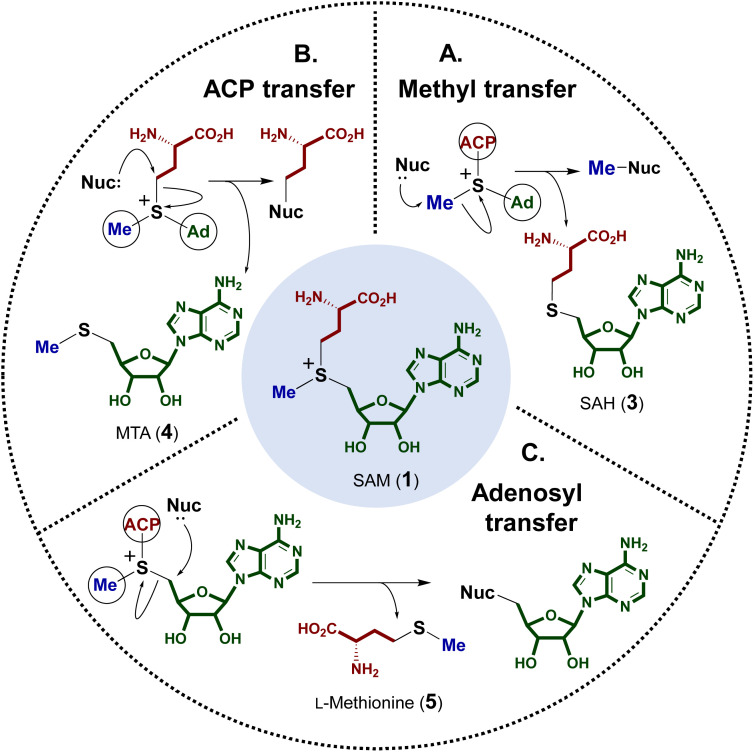
Summary of SAM-dependent alkylation reactions, showing the by-product generated following each group transfer. (A) Methyl transfer yields *S*-adenosyl homocysteine (SAH, 3), (B) ACP (3-amino-3-carboxypropyl) transfer yields 5′-methylthioladenosine (MTA, 4) and (C) adenosyl transfer yields l-methionine (5).

The electrophilicity of the SAM sulfonium moiety is illustrated by its involvement in a number of different biological alkylation reactions (Fig. 2). However, the regioselectivity of nonenzymatic alkyl transfer from sulfonium ions bearing three different primary alkyl groups cannot be precisely controlled by tuning either the steric bulkiness of the substituents or other reaction conditions such as the solvent, counter ion or polarizability.^25–28^ Consequently, the regioselectivity of enzyme catalyzed alkyl transfer from SAM appears to originate in variations in the enzyme active sites that tune the relative positioning between the alkyl donor and the nominal substrate, which serves as the alkyl acceptor. Moreover, a number of enzymes have also evolved active site arrangements that preferentially recognize derivatives of SAM thereby further increasing the scope of biological alkylation reactions. Protein crystallography has thus become a critical tool in understanding the details of SAM-dependent alkylation reactions. The following section is focused mainly on more recent findings regarding transfer of the 3-amino-3-carboxypropyl group and the modified methyl group from SAM, each catalyzed by an enzyme adopting a methyltransferase fold. Finally, a brief discussion is included of the adenosyl transfer enzymes, which lack a methyltransferase fold yet utilize SAM as the adenosyl donor.

### 2.1 SAM-dependent alkylation reactions

**Fig. 3:**
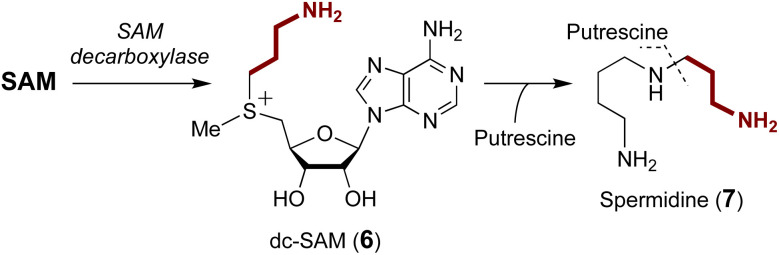
Aminopropyl group transfer during the biosynthesis of spermidine. SAM is first decarboxylated to form decarboxy-SAM (dc-SAM, 6) before the 3-aminopropyl group is transferred to putrescine to yield spermidine (7).

A number of enzymes have been discovered to selectively catalyze transfer of the 3-amino-3-carboxypropyl (ACP) group from the SAM sulfonium to an acceptor nucleophile expelling methylthioadenosine (MTA) as the leaving group. This type of chemistry has been described in the biosynthesis of several natural products as well as modification of tRNA and rRNA.^29–31^ Moreover, analogous reactions have also been reported for derivatives of SAM and in particular decarboxy-SAM (dc-SAM, 6) leading to aminopropyl transfer reactions observed during the biosynthesis of spermidine (7) (Fig. 3).^32–34^ Catalysis of both ACP transfer and aminopropyl transfer involves an inversion of the stereocenter consistent with a direct nucleophilic displacement reaction.^35–37^ Substrate binding is of particular importance for regioselective alkyl transfer, and thus the emphasis of the following discussion will be placed on how SAM binding allows for selective C–S bond cleavage.

#### 2.1.1 3-Amino-3-carboxypropyl transfer (small molecules)

**Fig. 4:**
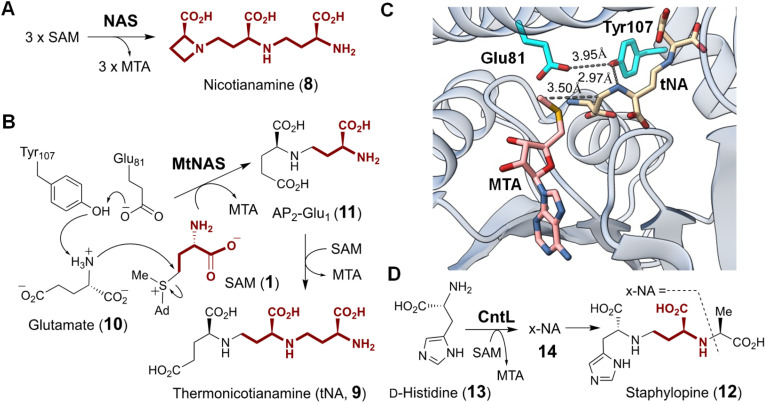
(A) Nicotianamine synthase (NAS) catalyzes the formation of nicotianamine (8) from three molecules of SAM. (B) Molecular mechanism proposed for MtNAS during the biosynthesis of thermonicotianamine (tNA, 9). (C) Crystallographic snapshot of MtNAS (PDB ID: 3FPF) complexed with MTA (pink) and tNA (tan). Nucleophilic displacement was facilitated by deprotonation of the substrate nitrogen by Tyr107 and Glu81 (cyan). (D) The NAS-like enzyme CntL catalyzes transfer of one ACP to d-His (13) providing x-NA (14) as the precursor of bacterial metallophore, staphylopine (12).

Nicotianamine (8) is a precursor to phytosiderophores, which facilitate metal acquisition by plants.^38,39^ Its biosynthesis involves nicotianamine synthase (NAS),^40^ which catalyzes the condensation of three ACP moieties each from one molecule of SAM and azetidine ring cyclization, though the timing of cyclization remains elusive (Fig. 4A).^41–44^ Although NAS had previously only been found in plants and fungi, recent studies have indicated that NAS-like enzymes exist in both archaea and bacteria as well.^45,46^

Archaeal NAS from *Methanothermobacter thermautotrophicus* (MtNAS) was found to copurify with thermonicotianamine (tNA, 9), which is structurally distinct from nicotianamine in that the first condensation partner is a glutamate (10) rather than an ACP moiety (Fig. 4B).^45^ Various crystallographic snapshots of MtNAS in complex with its substrate, a proposed intermediate and its product have led to a mechanistic proposal involving two rounds of ACP transfer to glutamate leading to tNA.^45,47^ In particular, the active site of MtNAS extends deep into the protein interior from a solvent exposed entrance allowing for sequential binding of glutamate and SAM.^45^ As SAM binds, glutamate translocates from the donor site at the entrance of the reaction chamber to the buried acceptor site. Transfer of the ACP moiety from SAM to glutamate or the intermediate AP_2_-Glu_1_ (11) is facilitated by Glu81 and Tyr107, which reside in the middle of the active site and deprotonate the nucleophilic amine on the acceptor substrate (*i.e.*, Glu or AP_2_-Glu_1_) (Fig. 4C).^45^

CntL is a bacterial NAS-like enzyme that participates in the biosynthesis of staphylopine (12),^46^ a metallophore in the pathogenic bacterium *Staphylococcus aureus*.^48–50^ Mutant strains that do not produce CntL show reduced virulence and decreased intracellular metal content.^46^ CntL catalyzes the transfer of ACP from SAM to d-histidine (13) forming xNA (14) (Fig. 4D).^46^ The structure of CntL was solved at 1.81 Å and found to be similar to that of MtNAS.^51^ Both MtNAS and CntL possess a Rossmann fold MT C-terminal domain with a characteristic glycine-rich motif that binds SAM as the ACP donor.^45,51^ An open to closed conformational change upon substrate binding was noted for CntL and involves the partially disordered interdomain linker in the CntL–SAM binary complex becoming an ordered α-helix in the CntL–MTA–xNA ternary complex.^51^ The mechanism of CntL catalyzed ACP transfer is otherwise analogous to that of MtNAS. In addition to NAS and CntL, many other NAS-like enzymes have been identified or proposed in the biosynthesis of structurally diverse metallophores with an amino group serving as the acceptor nucleophile, which underscores the importance of SAM-dependent ACP modification of metallophores in bacteria.^52–54^

**Fig. 5:**
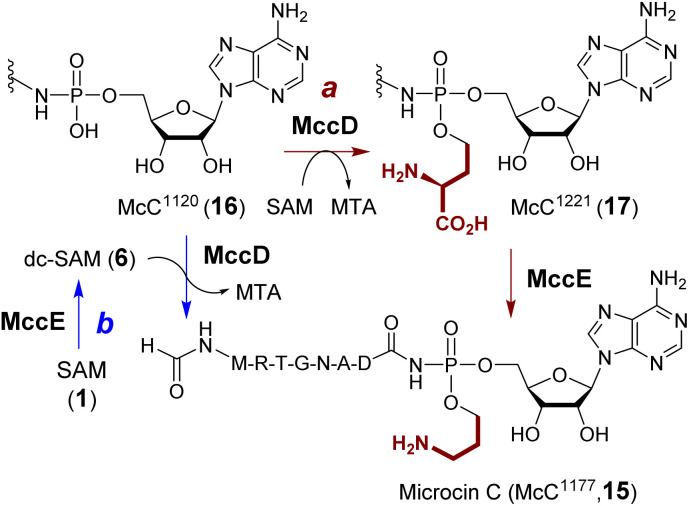
Aminopropylation during maturation of microcin C (15). MccD can catalyze transfer of an ACP group onto McC^1120^ (16), which can further be decarboxylated in a reaction catalyzed by MccE (route a). An alternative pathway starts with the formation of dc-SAM catalyzed by MccE followed by MccD mediated aminopropyl transfer (route b).

Microcin C (15) produced from *E. coli* is a potent inhibitor of aspartyl-tRNA synthetase.^55^ The biosynthesis of microcin C involves aminopropylation of the phosphate which enhances the inhibitory effect by approximately 10-fold.^56^ Two enzymes have been proposed to catalyze aminopropylation including a SAM-dependent methyltransferase MccD (PDB ID: 5FCD) and the N-terminal pyridoxal 5′-phosphate (PLP)-dependent decarboxylase domain of MccE (Fig. 5, route a).^56^*In vitro* characterization of the two enzymes initially suggested that aminopropylation may proceed with ACP transfer to McC^1120^ (16) catalyzed by MccD followed by decarboxylation of the alkylated intermediate McC^1221^ (17) catalyzed by MccE.^57^ However, an alternative route similar to polyamine biosynthesis, where decarboxy-SAM (6) is the aminopropyl donor such that MccE and MccD operate in the reversed order (Fig. 5, route b), has not been completely ruled out. Moreover, the activity of MccD can be significantly enhanced by the nucleosidase Mtn, which can hydrolyze the MTA by-product and presumably alleviate product inhibition.^57^

**Fig. 6:**
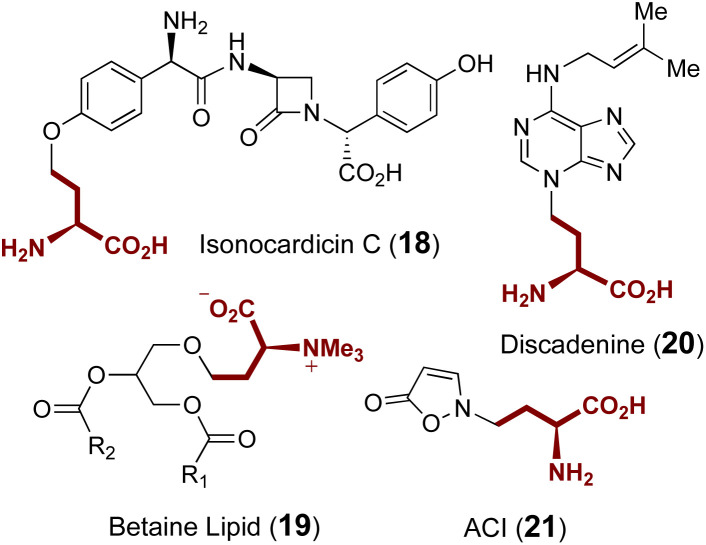
Other natural products with ACP groups derived from SAM.

Additional ACP transferases with small molecule substrates are also involved in the biosynthesis of isonocardicin C (18),^58,59^ betaine lipid (19),^60^ discadenine (20)^61,62^ and 2-(3-amino-3-carboxypropyl)-isoxazolin-5-one (ACI) (21) (Fig. 6);^63,64^ however, none of these enzymes appear to be homologous to the aforementioned ACP transferases NAS, MtNAS, CntL and MccD. With the exception of ACI, ACP transfer from SAM is respectively catalyzed in these cases by NAT, BtaA and discadenine synthase. However, while the *in vitro* formation of ACI is known to require a partially purified enzyme fraction from sweet pea seedlings as well as the presence of ATP and magnesium,^63,64^ the responsible enzyme as well as the role of ATP remains elusive.

#### 2.1.2 ACP transfer (RNA)

**Fig. 7:**
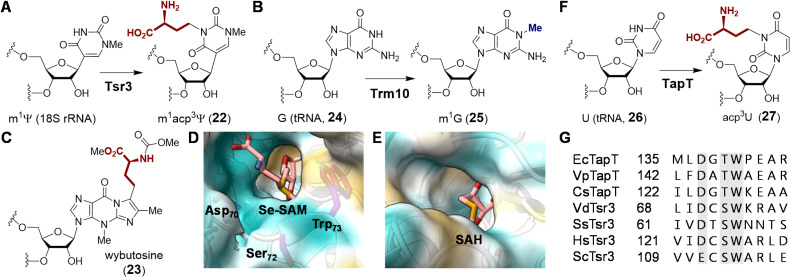
Enzymatic functions of (A) Tsr3 and (B) its closest homolog Trm10. Tsr3 catalyzes ACP modification of a pseudouridine residue whereas Trm10 catalyzes methylation of a guanosine or adenosine residue. (C) Structure of wybutosine highlighting the ACP moiety transferred under the action of Tyw2. Solvent accessible area of (D) Tsr3 (PDB ID: 5APG) and (E) Trm10 (PDB ID: 4JWF) is shown colored with the most hydrophilic residues in cyan to the most hydrophobic in gold. In Tsr3, the ACP side chain of Se-SAM (pink) is exposed to solvent and stabilized by hydrophilic interactions. Asp70, Ser72 and Trp73 in the DTW domain are in purple. In contrast, the ACP side chain of SAH (pink) in Trm10 is buried, and the entrance to the binding cavity is lined with hydrophobic residues. (F) Enzymatic function of TapT. (G) Sequence alignment of Tsr3 and TapT at the DTW domain (gray) (Ec: *Escherichia coli*; Vp: *Variovorax paradoxus*; Cs: *Clostridium saccharoperbutylacetonicum*; Vd: *Vulcanisaeta distributa*; Ss: *Sulfolobus solfataricus*; Hs: *Homo sapiens*; Sc: *Saccharomyces cerevisiae*).

Another class of ACP transferases is involved in the maturation of eukaryotic and archaeal 18S rRNA, where a uridine base (U1191 in yeast, U1248 in human) in the loop capping helix 31 undergoes a 3-step modification consisting of pseudouridylation, methylation and ACP transfer to yield 1-methyl-3-(3-amino-3-carboxypropyl)-pseudouridine (m^1^acp^3^Ψ, 22, see Fig. 7A).^65^ The participation of Tsr3 in ACP transfer was revealed by differences in the nucleoside profile of 18S rRNA isolated from a collection of mutant strains with random gene deletions.^66^ Crystal structures of archaeal Tsr3 from *Vulcanisaeta distributa* (VdTsr3) and *Sulfolobus solfataricus* (SsTsr3) have been solved with and without SAM-bound, respectively.^66^ The C-terminus of Tsr3 is closely packed with the N-terminal domain and adopts a SPOUT fold, which is characterized by a deep trefoil knot and often found in the structures of RNA methyltransferases.^67,68^ In contrast, its functional counterpart Tyw2 has a Rossmann fold and also catalyzes ACP transfer from SAM during the biosynthesis of wybutosine (23).^69^ Archaeal Tsr3 and its closest structural homolog Trm10,^70,71^ which is a tRNA guanidine *N*-methyltransferase (G, 24, to m^1^G, 25) found in archaeans and eukaryotes (Fig. 7B),^72^ employ a monomeric quaternary structure while the majority of enzymes in this superfamily are homodimers.^73^

Comparison of the crystal structures of VdTsr3 and Trm10 with SAM bound reveals overall similarity at the interface of the N- and C-terminal domains.^66^ This is where SAM binds with the adenine moiety buried in a hydrophobic pocket and the ribose moiety hydrogen-bonded to the protein backbone. However, closer inspection of the VdTsr3 active site shows the carboxyl moiety of ACP positioned towards the entrance of the substrate binding pocket surrounded by hydrophilic residues, while the methyl group is directed into a hydrophobic pocket consisting of an unconserved aliphatic residue and a conserved tryptophan (Trp73 in VdTsr3, see Fig. 7D).^66^ Mutation of the Trp to Ala results in both diminished SAM affinity and ACP transferase activity in SsTsr3.^66^ Furthermore, Tsr3 employs a conserved residue (Asp70 in VdTsr3) that serves as a general base, whereas the equivalent Asp210 in Trm10 is not essential for methyl transfer.^74^ The above catalytically important residues in Tsr3 constitute the DTW motif with the conserved primary sequence (D/E)X(T/S)W (Fig. 7G),^75^ in which Asp is the possible catalytic base and Trp assists in methyl group positioning.^76^ Proteins with this domain are classified into the TDD superfamily, which is named after the three representative protein members Tsr3, DTWD1 and DTWD2.^75^

Although ACP transfer catalyzed by Tsr3 is the final step in m^1^acp^3^Ψ modification during 18S rRNA maturation, studies of yeast mutants lacking enzymes for pseudouridylation and methylation have suggested that *in vivo* ACP transfer activity is independent of either modification such that the corresponding mutants can produce acp^3^U (27) or acp^3^Ψ, respectively.^77,78^ These results suggest that Tsr3 and its homologs can catalyze modification of unmodified uridine residues in RNA. Two recent studies involving comparative genomic and ribonucleome analysis have supported this hypothesis by showing that the bacterial Tsr3 homolog TapT from *E. coli* catalyzes acp^3^U (27) modification at U47 (26) in the V-loop of several bacterial tRNAs (Fig. 7F).^76,79^ While a structure of TapT is not yet available, sequence analyses and mutational studies imply that TapT harbors the catalytically important DTW motif similar to that of archaeal Tsr3 despite overall low sequence identity (Fig. 7G).^76^

#### 2.1.3 Carboxylmethyl transfer

**Fig. 8:**
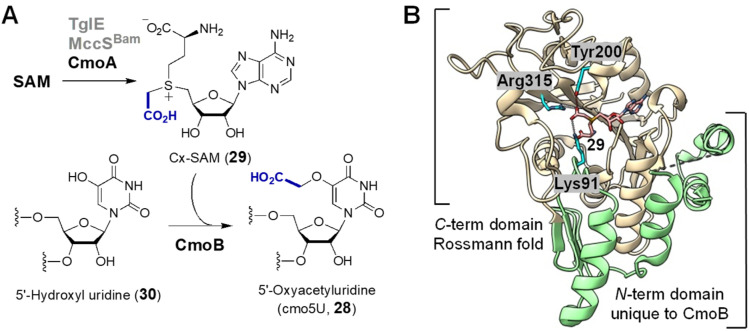
(A) Enzymatic functions of CmoA and CmoB. Two CmoA homologs TglE and MccS^Bam^ also catalyze the formation of 29. (B) Crystal structure of Cx-SAM bound to CmoB (PDB ID: 4QNV). The carboxymethyl moiety of Cx-SAM is coordinated by Tyr200, Lys91 and Arg315. The unique 100 residues at the N-terminus of CmoB are shown in green.

In Section 2.1.2, several SAM-dependent enzymes were described that catalyze transfer of the 3-amino-3-carboxypropyl (ACP) group to a nucleobase on tRNA thereby improving the thermostability and maintaining its normal function.^76,77,79,80^ 5′-Carboxymethyloxyuridine (cmo5U, 28), also known as 5′-oxyacetyluridine, is a modification on the wobble uridine of tRNA often observed in *E. coli* and many other Gram-negative bacteria that broadens recognition of degenerate codons during protein synthesis.^81–84^ Gene manipulation studies have suggested that formation of this unusual nucleobase involves the activity of two putative SAM-dependent methyltransferases CmoA and CmoB (Fig. 8A).^85^ CmoA catalyzes an unprecedented carboxylation of SAM yielding carboxy-SAM (Cx-SAM, 29),^86,87^ the details of which will be discussed in Section 2.5.1. Subsequently, CmoB transfers the carboxymethyl group from 29 to 5′-hydroxyl uridine (30) and generates 5′-carboxymethyloxyuridine (28).^86^

While CmoB can catalyze alkyl transfer from both SAM and Cx-SAM, it exhibits a strong preference for Cx-SAM.^88^ In order to identify the structural determinants of this selectivity, the crystal structure of CmoB was solved by Kim and co-workers (Fig. 8B).^88,89^ It was found that the C-terminal domain of CmoB adopts a Rossmann fold shared among typical methyltransferases, whereas the structure of the N-terminal domain, which spans around 100 amino acid residues, is unique to CmoB.^88^ Lys91 was found adjacent to the anionic carboxymethyl moiety and mutation of Lys to Ala completely abolished the production of cmo5U (28) *in vivo*, the effect of which is attributed to a 19-fold increase in affinity towards SAM and a 275-fold reduction in affinity towards Cx-SAM.^88^ Consequently, Lys91 likely serves to stabilize the carboxymethyl moiety in the active site *via* electrostatic interactions thereby enhancing the specificity of CmoB towards Cx-SAM (29) *versus* SAM. Furthermore, unlike typical SAM methyltransferases, CmoB does not appear to employ general base catalysis to increase the nucleophilicity of the acceptor oxygen,^88^ because the substrate hydroxyl has an apparent p*K*_a_ of 7.68 which is sufficient for specific acid/base catalysis.^90^

**Fig. 9:**
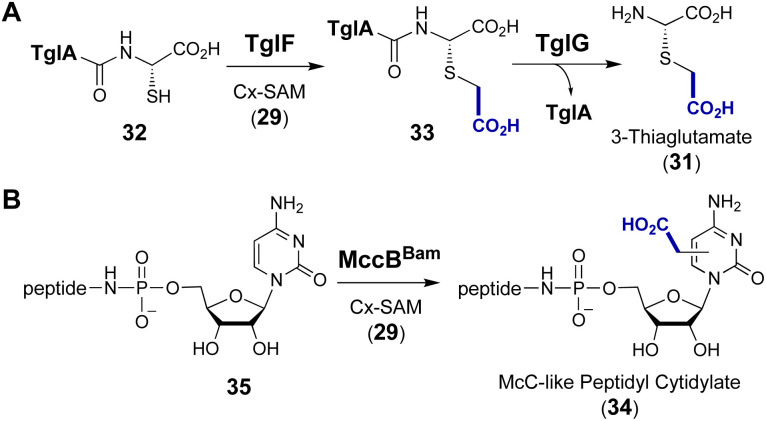
SAM-dependent carboxymethylation during the biosynthesis of (A) 3-thiaglutamate (31) and (B) McC-like peptidyl cytidylate (34).

Following discovery of the CmoA/CmoB carboxymethylation cascade, a homologous enzyme pair was found in the biosynthetic pathway of 3-thiaglutamate (31), a nonproteinogenic amino acid with unknown function.^91^ TglE/F show high sequence identity with CmoA/B, and TglF has been shown to catalyze the *trans*-carboxymethylation of a norcysteine thiol in a ribosomally synthesized and post-translationally modified peptide (32 → 33) prior to amide hydrolysis by the protease TglG to release 3-thiaglutamate (31) (Fig. 9A).^91^ Primary sequence alignment between CmoB and TglF showed that the essential residues including Lys91 for selective binding of Cx-SAM in CmoB are all conserved in TglF. Furthermore, the biosynthetic gene cluster (Mcc^Bam^) of the Trojan horse peptide-cytidylate antibiotic microcin C (34) encodes MccB^Bam^ which has been shown to catalyze transfer of the carboxymethyl moiety from Cx-SAM synthesized by MccS^Bam^ to its tRNA substrate 35 (Fig. 9B).^92^ The C-terminal domain of MccB^Bam^ (MccB^Bam^_CTD_) adopts a methyltransferase fold that does not show significant similarity with CmoB and is strictly specific for Cx-SAM (29).^92^ Although the site of carboxymethylation in 34 has yet to be determined, this finding implies that the two-enzyme cascade for this modification is operative in both Gram-negative and Gram-positive bacteria.^92^

The discovery of Cx-SAM demonstrates that SAM can be naturally modified leading to additional diversity among the available types of biological SAM-dependent alkyl transfer reactions. Given the high intracellular concentration of SAM *versus* its modified counterparts such as Cx-SAM,^88,93^ enzymes must employ recognition mechanisms in order to differentiate between the SAM congeners and thereby avoid inhibition or nonproductive shunt reactions. The structures of CmoB demonstrate that interactions between specific residues and the bound SAM analog at least play a part in this specificity. Consequently, it may be possible to engineer new alkyl transferases *via* optimized point mutations to facilitate the selective binding of other non-natural SAM analogs and thereby further expand the range of biocatalytic alkylation reactions for biotechnological purposes.^94,95^

#### 2.1.4 Adenosyl transfer

**Fig. 10:**
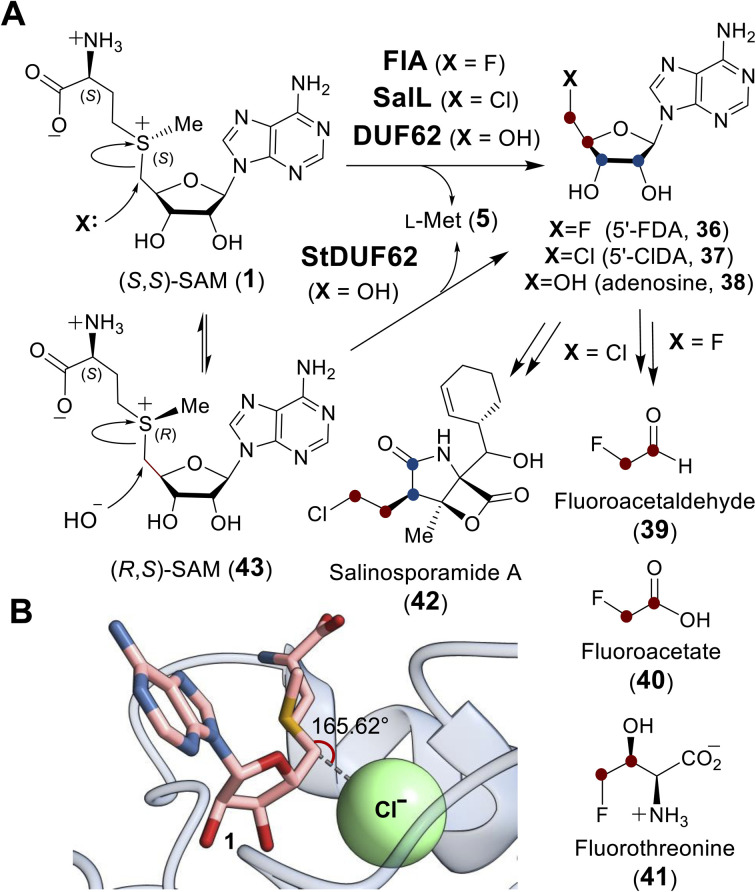
(A) Adenosyl transfer reactions catalyzed by FlA, SalL and DUF proteins. The resulting 5′-deoxy-5′-haloadenosine (36, 37) is a precursor to halogen containing natural products (39–42). (B) In the active site of SalL-Y70T mutant (PDB ID: 2Q6O), SAM (1, pink) and chloride (green) are aligned and poised for S_N_2 reaction.

Of the three possible alkyl transfer reactions involving SAM (Fig. 2), adenosyl transfer reactions are only known in the hydrolysis of SAM to methionine (5) and adenosine as well as the biosynthesis of 5′-deoxy-5′-haloadenosine.^96,97^ FlA from *Streptomyces cattleya* and SalL from *Salinispora tropica* are homologous enzymes sharing 35% sequence identity and catalyze adenosyl transfer from SAM to fluoride and chloride ions, respectively (Fig. 10A).^98–101^ The utilization of SAM in these biosynthetic pathways is not immediately obvious, because the resulting 5′-fluoro-5′-deoxyadenosine (5′-FDA, 36) and 5′-chloro-5′-deoxyadenosine (5′-ClDA, 37) are each metabolized by downstream enzymes before yielding the final halogenated natural products (39–42) with only part of the ribose skeleton from SAM remaining (Fig. 10A).^101,102^

**Fig. 11:**
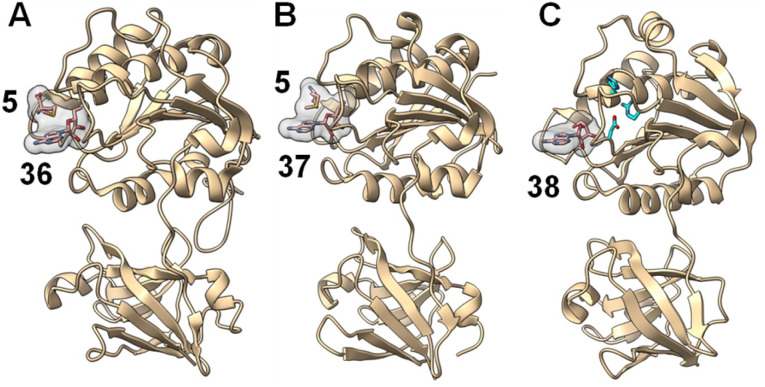
A similar overall structure was observed for three adenosyl transfer enzymes. (A) The product complex between fluorinase FlA/36/5 (PDB ID: 1RQR). (B) The product complex between chlorinase SalL/37/5 (PDB ID: 2Q6I). (C) The product complex of a DUF62 protein from *Pyrococcus horikoshii* OT3 with 38 (PDB ID: 1WU8). SAM-derived products (*i.e.*, 5 and 36–38) have a gray surface and the conserved His-Arg-Asp triad in DUF62 proteins is colored cyan.

Crystallographic studies of both FlA and SalL revealed an overall structure distinct from SAM-dependent methyltransferases. The structures of FlA and SalL instead resemble those of proteins belonging to the DUF62 superfamily despite none of the members having been functionally characterized at the time (Fig. 11). Subsequently, one protein from the DUF62 superfamily, SaDUF62, was shown to catalyze hydrolysis of SAM, which is effectively adenosyl transfer from SAM to a hydroxide ion thereby producing adenosine (38) and methionine (5).^103,104^ Moreover, a strictly conserved His-Arg-Asp triad is only present in DUF62 proteins (Fig. 11C), which activates one of the two H-bonded waters as the hydrolytic nucleophile necessary for the displacement reaction.^105^ In contrast, both halogenases lack the catalytic triad and waters are instead expelled from the vicinity of the sulfonium of SAM and the adjacently trapped halide nucleophile.

Comparison of the binary FlA/SAM substrate complex with the ternary FlA/5′-FDA/Met product complex has suggested that the C5′ of SAM is positioned between the fluoride and the sulfur of SAM during the substitution reaction indicative of an S_N_2-type mechanism.^100^ A catalytically inactive SalL mutant forms a ternary complex with chloride and SAM revealing a colinear arrangement between the chloride ion, C5′ and the sulfur center of SAM (Fig. 10B).^101^ Although the biological function of hydroxide adenosyltransferases is not as obvious as their halogenase counterparts, recent research identified a DUF62-containing protein, StDUF62, catalyzing the stereoselective hydrolysis of the non-native (*R*,*S*)-SAM (43), which is a stereoisomer that equilibrates with biological (*S*,*S*)-SAM (1) upon racemization of the sulfonium.^106^ This hints at a role played by DUF62 proteins in preventing *in vivo* accumulation of the nonbiological SAM diastereomer.

### 2.2 Intramolecular cyclization of SAM

**Fig. 12:**
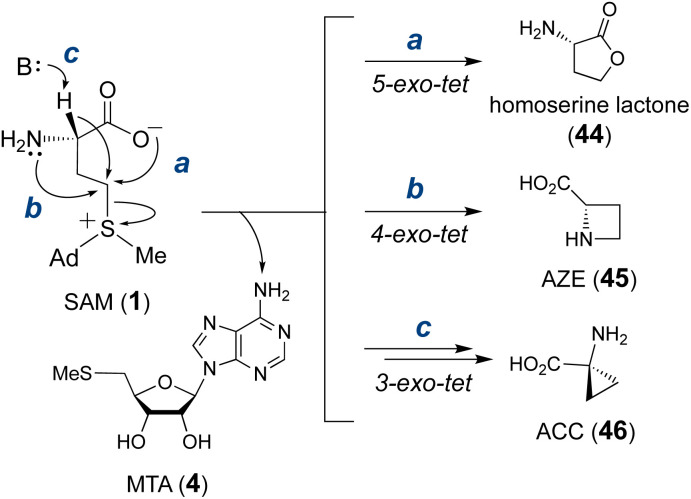
Intramolecular cyclization of the SAM aminocarboxypropyl moiety into homoserine lactone (44, *via* route a), azetidine 2-carboxylic acid (AZE, 45, *via* route b) and 1-aminocyclopropane-1-carboxylic acid (ACC, 46, *via* route c), each with elimination of MTA (4).

Transformations that utilize SAM as the alkylating agent take advantage of the inherent electrophilicity of the SAM sulfonium group^107^ This property renders SAM susceptible to nucleophilic attack not only intermolecularly but also intramolecularly. In fact, the major decomposition pathway of SAM under neutral or slightly acidic conditions is the intramolecular cyclization that yields homoserine lactone (44) and MTA (4) (Fig. 12).^108,109^ According to Baldwin's rules,^110^ this reaction is a favored 5-*exo*-tet ring closure and likely proceeds through direct nucleophilic attack of the carboxylate oxygen at the C-γ carbon. Although other non-enzymatic intramolecular cyclizations of SAM have not been reported, enzyme catalyzed cyclizations of the aminocarboxypropyl moiety to yield three-, four- and five-membered rings have been described in various natural product biosynthetic pathways (Fig. 12).^111–113^ However, not all of these biosynthetic enzymes adopt a SAM-dependent methyltransferase (MT) fold despite each utilizing SAM as the substrate. Therefore, only MT-like cyclases are included in the following section, and the rest will be discussed in Section 3.

#### 2.2.1 Azetidine 2-carboxylic acid

**Fig. 13:**
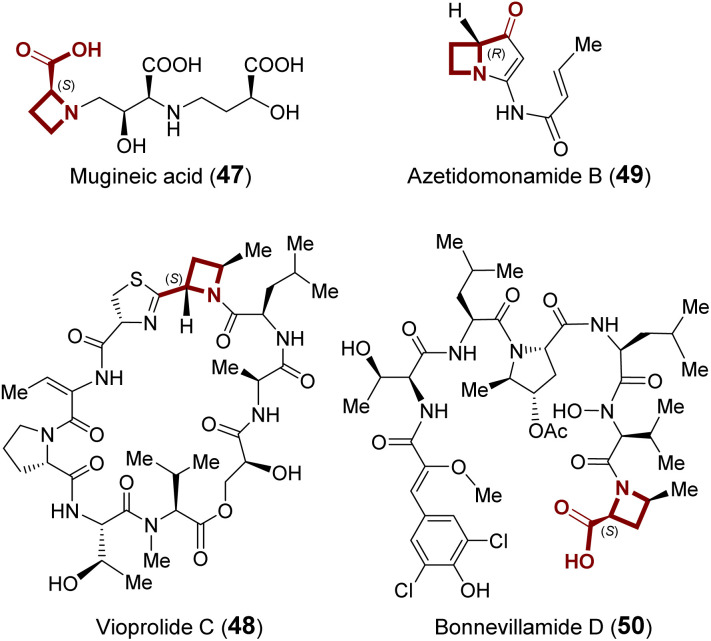
Examples of natural products that contain an azetidine 2-carboxylate (AZE) moiety.

Azetidine 2-carboxylic acid (AZE, 45) is a non-proteinogenic amino acid first isolated from *Convallaria majalis*.^114^ While it has been suggested that the four-membered ring was biosynthesized *via* intramolecular cyclization of aminocarboxypropyl of SAM, the details remained unelucidated.^115–117^ Since the discovery of AZE, several natural products carrying the AZE moiety have been isolated and their biosyntheses have been proposed to utilize SAM as the precursor to AZE, including nicotianamine (8) and the mugineic acid family of phytosiderophores (47).^118–120^ Recently, AZE formation during the biosynthesis of both vioprolide (48) and azetidomonamide (49) was independently reported by the Li and Müller groups.^121–123^ The responsible enzymes (*i.e.*, VioH and AzeJ) share only 28% sequence identity and catalyze the conversion of SAM to AZE (45). Similar chemistry has also been proposed for the homologous enzyme BnvI during the biosynthesis of bonnevillamides (50) (Fig. 13).^124^ Both AzeJ and VioH have been reported to display relatively low turnover numbers with the *k*_cat_ of AzeJ being 17 min^−1^ and that of VioH being too low to readily measure.^122,123^ Furthermore, VioH is most active under alkaline conditions indicative of general base catalysis to facilitate nucleophilic attack of the amino group at C-γ.^122^ However, none of these enzymes have been structurally characterized, and homology models have been inconclusive due to the lack of a suitable template.^122^ Consequently, it remains uncertain how the ACP side chain of SAM is arranged in the active site to facilitate the 4-*exo*-tet cyclization and avoid lactonization.

#### 2.2.2 Cyclopropane

**Fig. 14:**
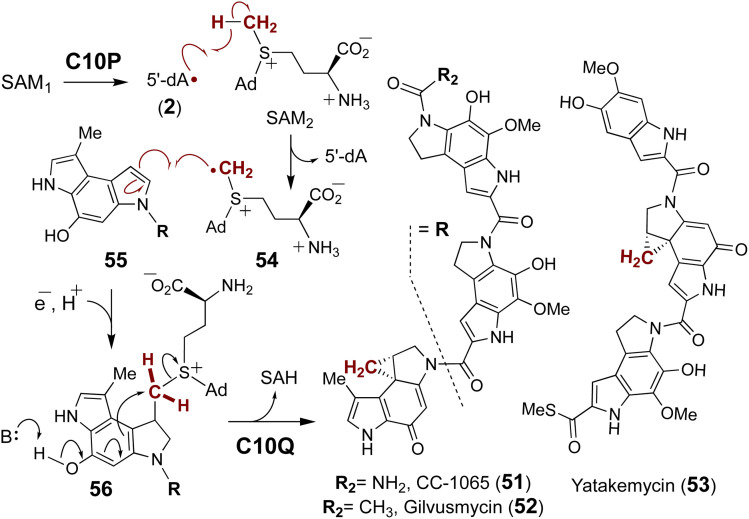
The proposed two enzyme cascade for C10P and C10Q catalyzed formation of the cyclopropane ring during biosynthesis of CC-1065 (51) and related compounds (52 & 53).

SAM-dependent two-enzyme cascades have recently been reported to install a spirocyclopropyl moiety in CC-1065 (51)^125^ and possibly gilvusmycin (52)^126^ and yatakemycin (53)^127^ as well (Fig. 14). CC-1065 biosynthesis involves the HemN-like radical SAM enzyme C10P and a SAM-dependent methyltransferase C10Q.^125^ In a coupled assay, the two enzymes together catalyze the cyclopropylation of 55 yielding CC-1065 (51), the mechanism of which was proposed to involve intermediate 56 generated from the addition of a SAM methylene radical (54) to the pyrrole ring of the nominal substrate (55) followed by reduction under the action of C10P. The subsequent cyclopropane ring formation is hypothesized to be catalyzed by C10Q and proposed to involve base catalyzed intramolecular elimination of SAH.^125^ The reaction proposed to be catalyzed by C10Q is analogous to other intramolecular elimination reactions involving SAM; however, in this case the methyl group has been modified with the moiety that serves as the nucleophile during the intramolecular cyclization.^125^ While this biosynthetic pathway is fully consistent with all available experimental data including detection of 56 by high resolution mass spectrometry and related isotope labelling results,^125^ direct characterization of C10Q with 56 in the absence of C10P has been hampered by poor stability of the latter.^125^ Therefore, many aspects of C10P/C10Q chemistry remain to be fully established.

### 2.3 Methylation induced cascade reaction

**Fig. 15:**
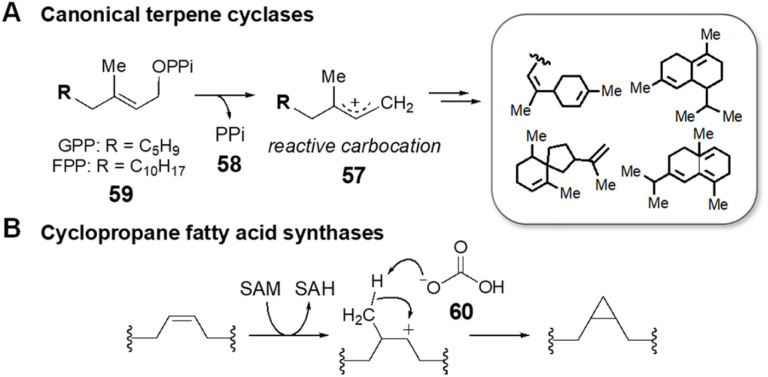
Cation-induced cyclizations catalyzed by (A) canonical terpene cyclases and by (B) cyclopropane fatty acid synthases.

SAM is also involved in enzyme catalyzed cyclization reactions that proceed in tandem with alkylation.^128,129^ The majority of examples of this chemistry characterized to date come from the study of terpene biosynthesis. Terpenoid lipids are characterized by extremely diverse cyclic scaffolds constructed from the two unsaturated precursors isopentenyl-pyrophosphate and dimethylallyl-pyrophosphate.^130,131^ The structural diversity is attributed to the formation of carbocation intermediates such as 57 that can rearrange in a number of ways within the active sites of terpene cyclases.^132,133^ The activated carbocation intermediates are typically generated *via* departure of pyrophosphate (58), which may still be associated with the carbocation species as an ionic complex, from the respective precursors (59) (Fig. 15A); however, synthetic methodologies have been developed that employ a Lewis acid to induce carbocation formation including epoxide ring opening and alkene protonation.^134^ This type of chemistry has also been found in natural product biosynthesis where SAM serves as the Lewis acid catalyst by essentially donating a methyl cation to the electron rich π-system of the terpenoid precursor. Cyclization of the resulting carbocation thus proceeds in tandem with alkylation before deprotonation, which in the case of cyclopropyl fatty acid synthases is performed by an active site bicarbonate ion (60) (Fig. 15B).^135,136^

#### 2.3.1 Terpene cyclization

**Fig. 16:**
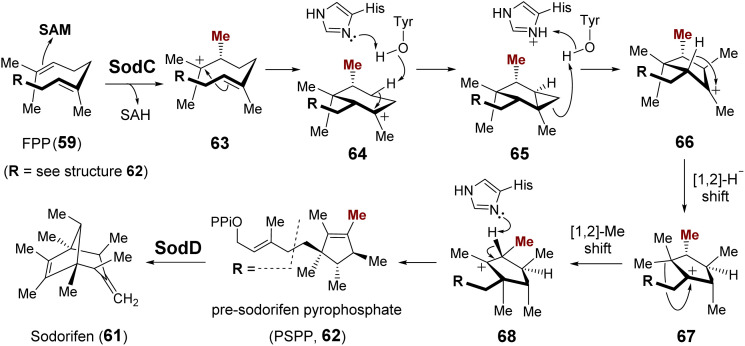
Mechanism proposed for the SodC catalyzed cyclization of FPP.

Sodorifen (61) is a 16-carbon terpene natural product isolated from *Serratia plymuthica*,^137^ which possesses the four-gene *sod* cluster responsible for sodorifen biosynthesis.^138,139^ Biosynthesis involves SodC catalyzed cyclization of farnesyl pyrophosphate (FPP, 59) to yield the biosynthetic intermediate pre-sodorifen pyrophosphate (PSPP, 62), which is subsequently converted to sodorifen under the action of SodD (Fig. 16).^139,140^ The tertiary structure of SodC was predicted by *in silico* homology modeling, which allowed for further computation of the reaction mechanism.^140^ It has thus been proposed that PSPP formation proceeds through a cyclopropane intermediate (65) that is generated upon the carbocationic rearrangement of FPP following SodC catalyzed π-methylation by SAM (59 → 63).^140^ The initially generated cyclohexane carbocation first undergoes ring contraction in a stepwise process involving a neutral bicyclic intermediate (64 → 65 → 66).^140^ The resulting carbocation 66 can then undergo additional hydride and methyl shifts directed by an active site Tyr-His dyad leading to PSPP formation with an overall release of energy.^140^ Bioinformatics analysis of clustered *sodC* and *sodD* homologs has identified 28 *sod*-like biosynthetic gene clusters that may also encode additional methyltransferases, Rieske proteins and flavodoxin proteins.^141^ This suggests a number of additional exotic terpenoid ring systems remain to be discovered.

#### 2.3.2 Pictet–Spengler rearrangement

**Fig. 17:**
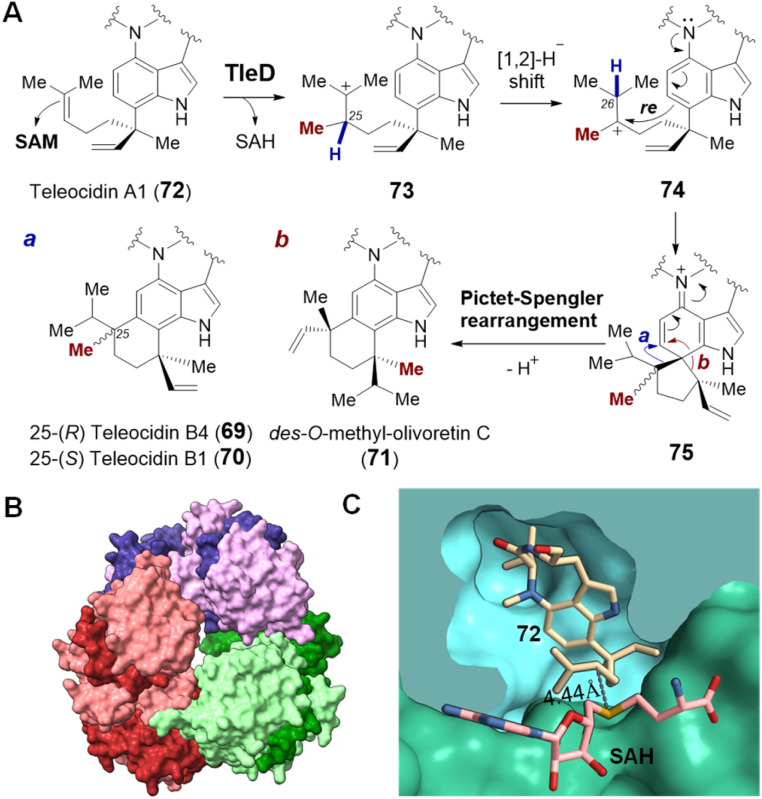
(A) Methylation induced cyclization catalyzed by TleD in teleocidin biosynthesis. Crystal structures of (B) TleD hexamer (PDB ID: 5GM2) and (C) TleD active site formed at the interface of two monomers (green & blue) with bound teleocidin A1 (72, tan) and product SAH (pink).

Another example of an alkylation induced carbocationic rearrangement and cyclization comes from the biosynthesis of teleocidins (69–71).^128,142,143^ TleD is a SAM-dependent methyltransferase that catalyzes C25 methylation of the isobutenyl moiety of teleocidin A1 (72) to generate a putative tertiary carbocation intermediate (73, Fig. 17A). As in the case of terpene biosynthesis, the resulting carbocation may then undergo a [1,2]-hydride shift (73 → 74), which is consistent with deuterium incorporation experiments, followed by intramolecular electrophilic aromatic substitution to yield an intermediate containing an unstable spiro-ring system (75).^144^ Subsequent Pictet–Spengler rearrangement^145,146^ and deprotonation would then yield 69–71.

Crystal structures of TleD have been solved with SAH bound (2.5 Å) as well as both SAH and substrate bound (2.8 Å) in the active site.^147^ These structures place the sulfur of SAH approximately 4.4 Å away from C25 of the teleocidin A1 (72), which is consistent with the proposed methyltransfer mechanism.^147^ Unlike typical SAM methyltransferases, TleD appears to be active as a hexamer with each active site formed at the interface of two TleD monomers in a domain-swapped fashion (Fig. 17B and C).^147^ The active site of TleD is tightly lined with hydrophobic residues and thus water is excluded and prevented from quenching the putative intermediate carbocations.^147^ The geranyl moiety of the substrate appears to be flexible and assumes two different conformations in the crystal structure; however, molecular dynamics analysis suggests that only one of them is likely to account for the observed product distribution.^147^

#### 2.3.3 Stevens rearrangement

**Fig. 18:**
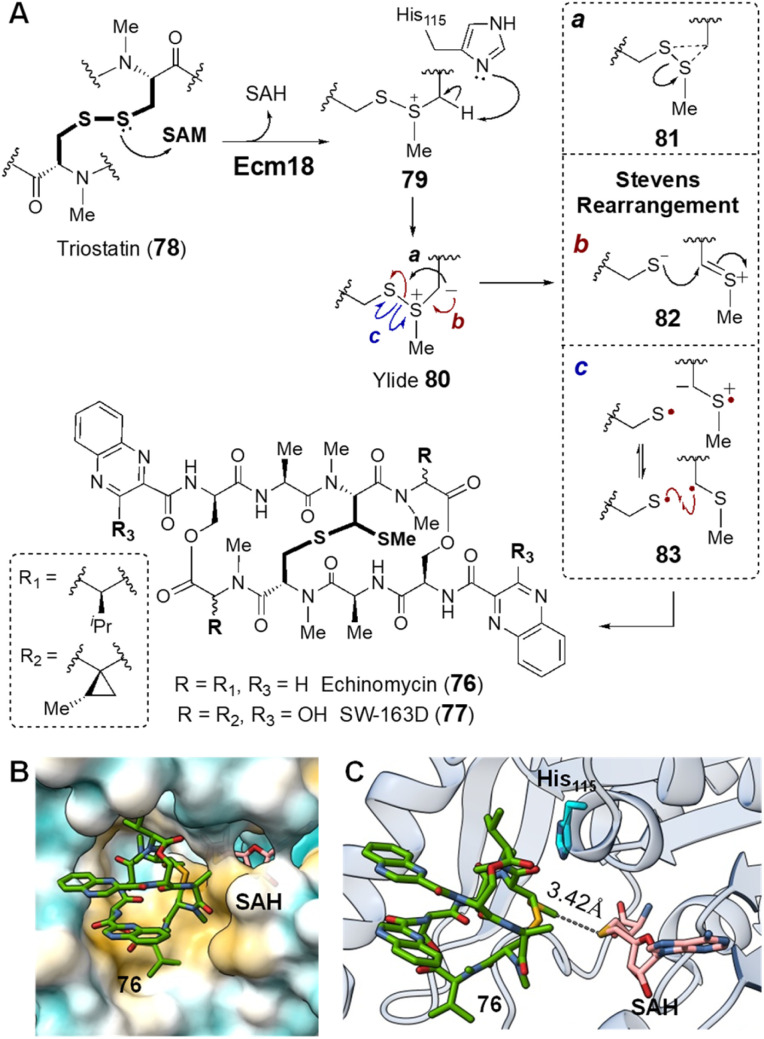
(A) Mechanism of Ecm18 catalyzed rearrangement of a disulfide bridge to a thioacetal. (B) Hydrophobic surface of the Ecm18 substrate binding pocket (PDB ID: 4NEC) colored according to most hydrophobic (gold) and most hydrophilic (cyan) residues. (C) Ecm18/76/SAH ternary complex. The distance between the sulfur of SAH and the methyl group in 76 is consistent with the proposed methyltransfer reaction. The basic nitrogen on His115 (cyan) is near the disulfide bridge and could facilitate ylide 80 formation *via* deprotonation of the methylated sulfonium intermediate 79.

Alkylation induced carbocationic rearrangements can also be initiated *via* the methylation of moieties other than alkenes and do not necessarily need to involve cyclization. For example, this type of chemistry appears to be relevant to the biosynthesis of echinomycin (76), which is also known as quinomycin A and is a nonribosomal peptide antiproliferative and antibiotic agent.^148–150^ The structure of echinomycin features a methyl thioacetal linkage, which is introduced *via* a redox-neutral rearrangement of the disulfide bridged precursor triostatin (78)^151^ and is believed to lend additional stability to the dimer compared to its disulfide counterpart (Fig. 18A).^152,153^ The enzyme responsible for this transformation is the SAM-dependent methyltransferase Ecm18 (Qui8)^154–156^ or Swb8 in the case of the related compound SW-163D (77) and its derivatives.^157,158^

The catalytic cycle of Ecm18 is hypothesized to proceed through an intermediary sulfonium ion generated *via* SAM-dependent methylation of one of the two sulfur atoms of the disulfide bridge in triostatin (78 → 79).^154^ The sulfonium ion can then undergo α-deprotonation in the presence of a general base to yield an ylide (80), which is capable of a Stevens rearrangement to yield the methylthioacetal linkage (80 → 76). To probe the mechanistic details of Ecm18 catalysis, Hotta and co-workers solved the 1.5 Å crystal structure of Ecm18 with the reaction products SAH and echinomycin (76) bound in the active site.^159^ Ecm18 was thus found to adopt a characteristic SAM binding Rossmann fold featuring a compact hydrophobic substrate/product binding site (Fig. 18B).^159^ The proposed methylation reaction is consistent with the linear arrangement of the sulfur center of SAH, the transferred methyl group and the sulfur center on the product. A polar histidine residue resides sufficiently close to the thioacetal and could serve as a general base for α-deprotonation of the substrate sulfonium to yield the putative sulfur ylide 80 (Fig. 18C).^159^ Rearrangement of the ylide may then proceed *via* a 3-*endo*-tet cyclization and ring-opening to yield the methyl thioacetal in 76 (Fig. 18A, mechanism a).^159^ However, two other mechanistic hypotheses for the rearrangement have also been proposed that do not require intermediary cyclization *via*81 to effect the Stevens rearrangement as shown in Fig. 18A.^107,160,161^ One involves heterolytic cleavage of the S–S bond followed by thiolate addition to the resulting thionium 82 (mechanism b). The other starts with homolytic cleavage of the S–S linkage and a subsequent radical–radical recombination to construct the C–S bond in 76 (mechanism c).

#### 2.3.4 Alkaloid cyclization

**Fig. 19:**
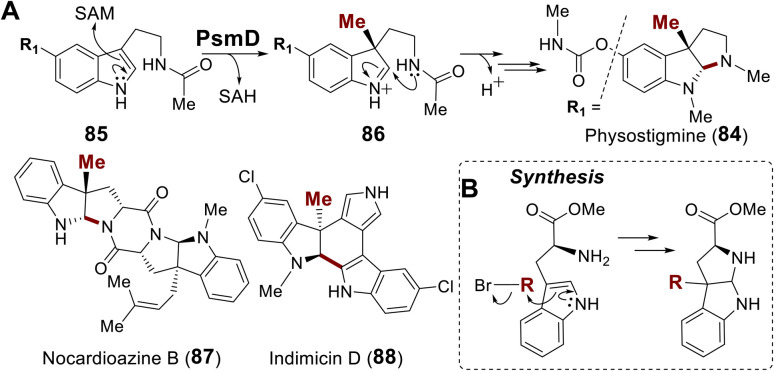
(A) Methylation induced cyclization in pyrroloindoline alkaloid biosynthesis. (B) Synthesis of pyrroloindolines *via* alkylation–cyclization cascade.

Biosynthesis of a subclass of pyrroloindoline alkaloids has also been shown to involve an alkylation–annulation cascade.^162^ Natural products in this family such as physostigmine (84) are characterized by complex, fused ring systems derived from tryptophan.^163^ Liu and co-workers discovered that the SAM-dependent methyltransferase PsmD is an early participant in the biosynthesis of physostigmine where it catalyzes cyclization of 85.^164^ PsmD catalysis begins with methylation at C3 of the indole moiety resulting in an iminium ion 86 that undergoes intramolecular nucleophilic addition by a nearby amide nitrogen to afford the heterocycle in 84 (Fig. 19A).^164^ A similar methylation induced cyclization has been reported in the biosynthesis of nocardioazine B (87) and proposed in indimicin (88).^165,166^ Evidence for such chemistry comes from model systems, which have demonstrated that C3 alkylation of the indole moiety always results in intramolecular addition of the adjacent amide.^167,168^ Thus, only the alkylation step appears to require enzyme catalysis with subsequent cyclization following nonenzymatically. Such alkylation–annulation tandem reactions have also been utilized in the total synthesis of an expanded scope of alkylated pyrroloindoline scaffolds in which alkyl bromides are used as the alkyl donor (Fig. 19B).^168^

### 2.4 SAM as a prosthetic group

Apart from direct participation in the group transfer reactions, SAM can also serve as a prosthetic group for many enzymes that are annotated as SAM-dependent methyltransferases. Among these examples, SAM does not undergo any covalent changes in its structure during the reaction.

#### 2.4.1 LepI in leporin biosynthesis

**Fig. 20:**
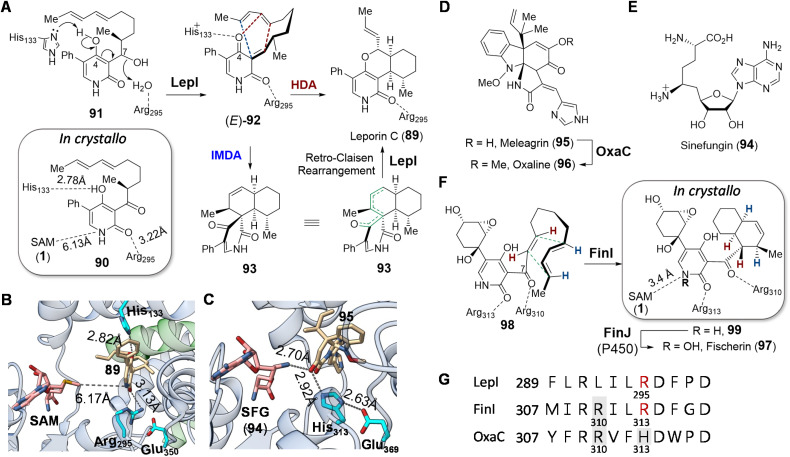
(A) LepI catalyzed dehydration–cyclization cascade during leporin C biosynthesis. Although the stereochemistry at C7 in 91 is currently unclear, it has been shown that the diastereomer of 91 is not accepted by LepI. Comparison of crystal structures of (B) LepI/SAM/89 complex (PDB ID: 6IX9) and (C) OxaC/SFG/95 complex (PDB ID: 5W7S). In LepI, SAM is too far from the product (89, in tan) for methyl transfer; however, in the methyltransferase OxaC, the substrate meleagrin (95) (tan) is located in between SFG and the His313-Glu369 dyad. In contrast, the catalytic histidine is substituted with Arg295 in LepI such that deprotonation cannot occur. Green ribbons in LepI are residues from the second monomeric peptide. (D) OxaC catalyzed methylation during formation of oxaline (96). (E) Structure of sinefungin (94). (F) Cyclization of 98 catalyzed by FinI. (G) Sequence alignment of LepI, FinI and OxaC highlighting the conserved SAM binding arginine residue (Arg310) that interacts with SAM, the catalytic base (His313) in OxaC (in gray boxes) and the essential arginine (Arg295/Arg313) in the pericyclases in red.

Leporins (89) are pyridone alkaloids exhibiting mild antibacterial activity.^169^ Within the *lep* biosynthetic gene cluster, LepI is annotated as a SAM-dependent *O*-methyltransferase and contains a C-terminal Rossmann fold for SAM binding.^170^ However, *in vitro* analysis suggests that LepI accelerates a stereoselective dehydration reaction of its nominal substrate (91, the stereochemistry of the carbon with the departing hydroxyl group is not yet known) to (*E*)-92, and facilitates a subsequent regioselective hetero-Diels–Alder (HDA) reaction (92 → 89) (Fig. 20A). Meanwhile, LepI can also convert a shunt intramolecular-Diels–Alder (IMDA) product 93 to 89*via* a retro-Claisen rearrangement.^170^ Consistent with its annotation, however, catalytically active LepI copurifies with SAM at almost 90% occupancy. Furthermore, sinefungin (SFG, 94), which is an isosteric analog of SAM, also supports LepI catalysis, while SAH (3) is a strong inhibitor of both the dehydration and retro-Claisen rearrangement. Therefore, the positive charge on SAM or sinefungin appears to play a key role in LepI catalysis even if SAM itself remains intact.^170^

**Fig. 21:**
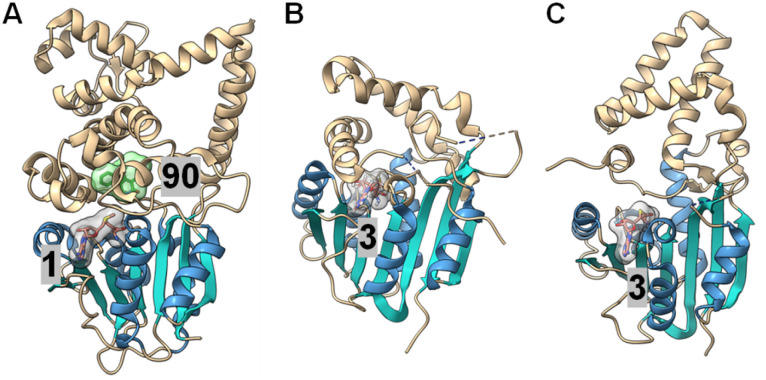
Crystal structures of (A) LepI (PDB ID: 6IX5), (B) SpnF (PDB ID: 4PNE) and (C) SpnL (PDB ID: 7V6H). Each enzyme contains a C-terminal Rossmann fold catalytic domain containing seven β-strands (cyan) surrounded by five α-helices (blue), while the N-terminal domains differ between each protein. The cofactor, SAM (1) for LepI and SAH (3) for SpnF and SpnL (pink with a gray surface), binds similarly at the C-terminal domain of all three enzymes. The substrate analog (90) bound in the LepI active site is shown with a green surface.

The positive charge on SAM and sinefungin was originally proposed to play an electrostatic role in catalysis, since coordination of the substrate to a sulfonium moiety is suggested by computational simulations to accelerate the cyclization and drive the periselectivity towards the favored product.^170^ However, the crystal structure of LepI complexed with SAM and an unreactive substrate analog (90) revealed that the distance between the two ligands is too remote for coordination (Fig. 20A and 21),^171^ whereas a product complex between LepI/SAM/89 indicated that the guanidinium group of Arg295 forms a hydrogen bond with the amide carbonyl of the LepI product (89).^171^ In addition, the His133 residue essential for LepI catalysis is near the 4-O of 89, implying its role in deprotonation of 91 (Fig. 20B). The resulting imidazolium of His133, along with the guanidinium of Arg295, is thus hypothesized to stabilize the transition-state during cycloaddition *via*92.^171^ This conclusion has also been corroborated by three other independent crystallography studies.^172–174^ Sequence and structural comparison between LepI and its closest homolog OxaC, which is an *O*-methyltransferase catalyzing the methyltransfer of meleagrin (95) to yield oxaline (96) (Fig. 20D),^175^ revealed that Arg295 in LepI aligns with an essential histidine (His313) in OxaC (Fig. 20G).^171^ His313, along with Glu369, is conserved among functional *O*-methyltransferases for substrate deprotonation, and the substitution of the former in LepI may provide an evolutionary perspective on the mechanistic shift from performing methylation to dehydration and cyclization.

Such repurposed methyltransferases catalyzing cyclization reactions have also been found to participate in other biosynthetic pathways. For example, fischerin (97) features a *cis*-decalin moiety, the biosynthesis of which is not immediately obvious. The dedicated cyclase FinI shows 25% sequence identity and structural homology with LepI and also co-purifies with SAM as does LepI.^170,176^ Moreover, FinI catalyzed cyclization of 98 to 99 is believed to involve an *exo*-selective DA reaction (Fig. 20F), which is rare among known pericyclases.^176^ Unlike the case of LepI, however, SAM and the substrate 98 bind the FinI active site so as to place the sulfonium methyl of SAM close to the amide nitrogen of 98 at a distance of 3.4 Å suggesting an electrostatic interaction and a possible role for SAM in substrate recognition. Whereas in the case of LepI Arg295 can act as a Lewis acid to activate the diene for cyclization,^171^ the homologous residue Arg313 in FinI is unable to do so given the structure of 98 (Fig. 20F). Instead, Arg310 may play a similar role serving to activate the dienophile in the FinI catalyzed reaction by forming a hydrogen bond with the carbonyl group at C7 of 98. Although Arg310 in FinI is conserved in homologous methyltransferases such as OxaC, where it interacts with SAM, this residue appears to have been evolutionarily repurposed in the FinI active site to interact and likely activate the substrate for cyclization (Fig. 20G).

#### 2.4.2 SpnF and SpnL in spinosyn biosynthesis

**Fig. 22:**
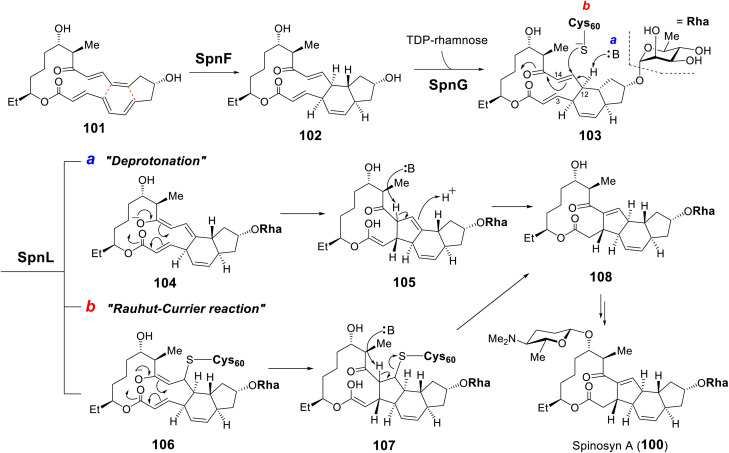
Biosynthesis of spinosyn A highlighting the [4 + 2] cycloaddition catalyzed by SpnF and two proposed mechanisms for the transannulation catalyzed by SpnL.

SpnF and SpnL participate in the biosynthesis of the tetracyclic polyketide spinosyn A (100) and are both annotated as SAM-dependent methyltransferases with a SAM binding Rossmann fold at the C-terminal;^177^ however, neither enzyme catalyzes a methyl transfer reaction. SpnF instead catalyzes an intramolecular [4 + 2]-cycloaddition (101 → 102), whereas SpnL catalyzes the cyclization of 103, yielding the first biosynthetic intermediate 108 with the same ring skeleton as spinosyn A.^177^ The catalytic properties of SpnF were identified when an approximately 500-fold increase in reaction rate was observed for the cyclization reaction in the presence of the enzyme *versus* its absence (Fig. 22).^177^ Furthermore, measurement of secondary kinetic isotope effects (KIE) suggested that both non-enzymatic and SpnF-catalyzed cyclization likely proceed *via* a stepwise rather than a concerted mechanism,^178^ in agreement with computational studies.^179,180^ In contrast to LepI, however, active SpnF copurifies with SAH rather than SAM.^181^ The crystal structure of the SpnF–SAH binary complex was solved at 1.50 Å (Fig. 21B), and computational docking of substrate or product into the hydrophobic cavity of SpnF implied that neither substrate nor product exhibits direct contact with the SAH moiety.^181^ Therefore, SAH may serve as a component necessary for the integrity of the SpnF structure, and the positive charge of the sulfonium ion of SAM is less likely to be essential for catalysis.^181^

**Fig. 23:**
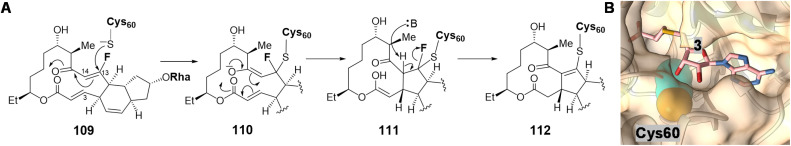
(A) C13-fluorinated substrate (109) leads to covalent modification of SpnL. (B) Crystal structure of SpnL/SAH binary complex (PDB ID: 7V6H) highlighting the SAH (3) binding site and the catalytic Cys60 (carbons as cyan and sulfur as yellow spheres), which is buried in the protein interior.

Following SpnF catalyzed [4 + 2] cycloaddition and SpnG catalyzed glycosylation,^182^ SpnL catalyzes the transannular carbon–carbon bond formation between C3 and C14 of 103 to generate 108 (Fig. 22).^177^ Two possible mechanisms have been postulated, the first involving deprotonation at C12 to form an enolate (104) poised for Michael addition at C3 giving 105. Alternatively, a nucleophilic residue in the active site may first add to C13 to yield intermediate 106, which may then cyclize into 107 prior to elimination of the assisting nucleophile. Involvement of a nucleophilic residue during modification of the β-carbon of an enone moiety is indicative of a Rauhut–Currier (RC) reaction^146,183^ and is reminiscent of thymidylate synthase chemistry.^184^ When the SpnL reaction was conducted in buffered D_2_O, only a single solvent deuterium was incorporated into the product; however, no significant primary KIE was observed with the C12-deuterated substrate.^185^ Moreover, a C13-fluorinated substrate analog 109 led to inactivation of SpnL *via* the formation of a covalent enzyme–substrate adduct (112) demonstrated by mass spectrometry (Fig. 23A).^185^ Thus, all the biochemical evidence is consistent with an RC mechanism. SpnL shares 35% sequence identity with SpnF and also copurifies with bound SAH.^185^ The binary complex of SpnL with SAH bound was solved at 3.05 Å (Fig. 21C) and demonstrated that Cys60 resides nearby the bound SAH with its thiol moiety buried and apparently inaccessible from the active site cavity (Fig. 23B).^185^ Consequently, a conformational change upon substrate binding was proposed to allow for Cys60 exposure to the substrate thereby facilitating catalysis.

**Fig. 24:**
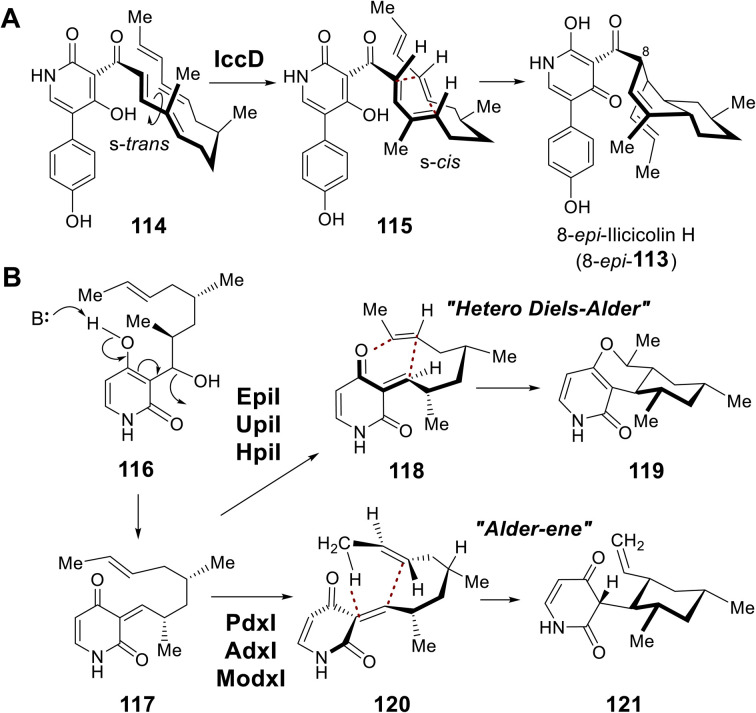
(A) Cyclization catalyzed by IccD during the biosynthesis of ilicicolin H. (B) Dehydration–cyclization reactions catalyzed by SAM-independent pericyclases that each adopt a SAM-binding fold.

During biosynthesis of the fungal secondary metabolite ilicicolin H (113), the cyclohexene moiety is believed to be constructed *via* an inverse-electron demand Diels–Alder (DA) reaction catalyzed by IccD, which harbors an intact SAM-binding motif yet does not copurify with SAM (Fig. 24A).^186^ Moreover, two additional groups of methyltransferase-like proteins were also found to catalyze pericyclic reactions. EpiI, UpiI and HpiI are proposed to catalyze a hetero-DA reaction yielding 119 on the quinone methide substrate (117) generated upon dehydration of 116, while PdxI, AdxI and ModxI catalyze conversion of the same substrate to 121*via* a proposed Alder-ene reaction (Fig. 24B).^187^ The catalytic cycles of these pericyclases, however, do not depend on either SAM or SAH indicating they are mechanistically distinct from those of LepI or SpnF and SpnL.^186,187^ Pericyclases characterized by a SAM-binding fold appear both evolutionarily and catalytically distinct.^188–190^

#### 2.4.3 SlnM in salinomycin biosynthesis

**Fig. 25:**
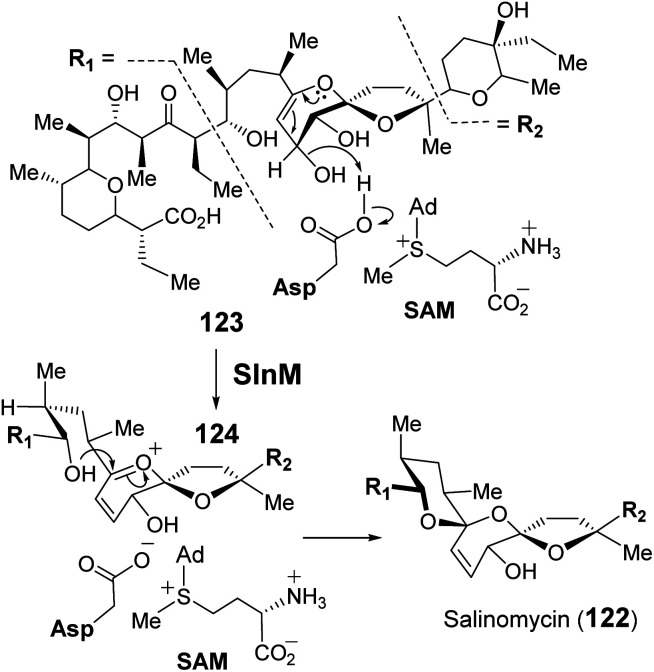
SlnM catalyzes spirocyclization during salinomycin biosynthesis. SAM is proposed to stabilize an anionic aspartate residue *via* electrostatic interactions to facilitate substrate protonation.

Salinomycin (122) is a broadly utilized coccidiostat featuring a bis-spiro tricyclic structure with a polyether linkage.^191^ Its derivatives have recently drawn attention in the field of oncology due to their antitumor potential.^192,193^ Within the biosynthetic gene cluster, SlnM is annotated as a SAM-dependent *O*-methyltransferase containing a glycine-rich motif canonical for SAM binding.^194,195^ Gene inactivation of SlnM results in the accumulation of a ring-opened intermediate (123) suggesting that SlnM catalyzes the dehydration and subsequent spirocyclization of the putative precursor species 124 (Fig. 25).^196^ The presence of SAM is essential for SlnM activity, though it is not consumed over the course of the reaction, and SAM can be replaced by its isosteric analog sinefungin (94) but not SAH.^196^ Consequently, SAM likely serves to both help maintain the structural integrity of SlnM and also contribute directly to catalysis in some manner that requires the positively charged sulfonium moiety. On the basis of sequence alignment, mutational studies and computational modelling, Asp58 and Asp186 were identified as catalytically important residues, with one presumably serving as the general acid that facilitates the dehydration reaction.^196^ It has therefore been hypothesized that the sulfonium of SAM facilitates elimination of water by stabilizing the negative charge buildup on the aspartate residues as they protonate the leaving group (Fig. 25).^196^ However, the exact positioning between SAM, substrate and these Asp residues has yet to be fully established due to the lack of a crystal structure.

#### 2.4.4 SAM-dependent hydroxylase

**Fig. 26:**
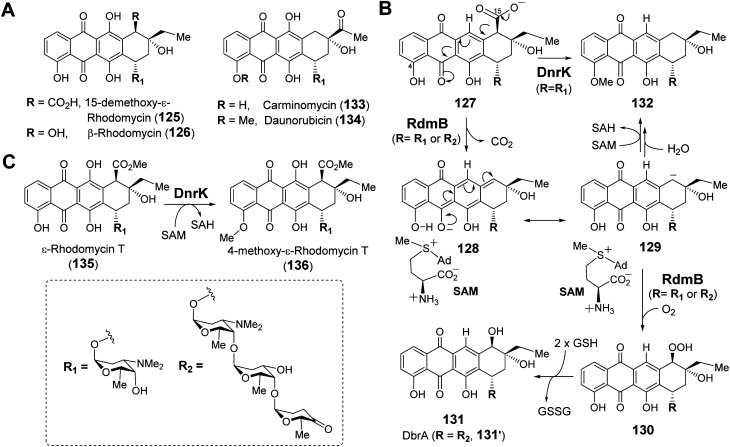
(A) Selected anthracycline natural products generated *via* RdmB or DnrK catalysis. (B) Proposed mechanism for RdmB/DnrK reaction with 127. (C) Activity of methyltransferase DnrK with 135.

The first example of a SAM-dependent hydroxylase is RdmB, which catalyzes decarboxylative hydroxylation of 15-demethoxy-ε-rhodomycin (125) to form the anthracycline precursor β-rhodomycin (126).^197^ While RdmB requires SAM for activity, it operates as an O_2_-dependent oxidase that recognizes several different substrates such as 127 in addition to 125 (Fig. 26A and B).^197–201^ These substrates all feature a carboxylate moiety at C10, which upon decarboxylation forms the anion 128 that is stabilized by extended conjugation. Moreover, sinefungin (94) but not SAH (3) is able to support hydroxylase activity, which led to the hypothesis that electrostatic stabilization of the anionic intermediate 128 by SAM is essential for catalysis.^200^ The intermediate 129 can then activate molecular oxygen to yield the peroxide 130. In the presence of reduced glutathione (GSH), 130 is converted to the hydroxylation product 131.

**Fig. 27:**
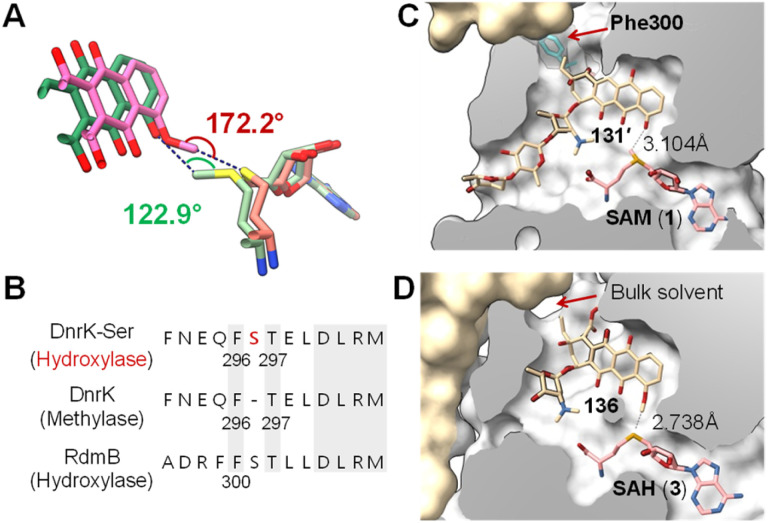
(A) Comparison of the binding of SAM (light green)/131′ (green) in RdmB and SAH (salmon)/136 (pink) in DnrK. (B) Sequence alignment of DnrK and RdmB at the α-helix that contains the aromatic residue, highlighting the position of serine insertion in DnrK-Ser. The orientation of Phe296 in DnrK or Phe300 in RdmB dictates the catalytic activity being methylation or hydroxylation. (C) Clip view of the RdmB/SAM/131′ complex (PDB ID: 1XDS). (D) Clip view of the DnrK/SAH/136 complex (PDB ID: 1TW2). The channel to the bulk solvent is blocked in RdmB due to the presence of Phe300 (cyan), whereas it is opened in DnrK.

There is reason to believe that RdmB may have diverged from an evolutionary lineage of SAM-dependent *O*-methyltransferases upon losing methyltransferase activity. DnrK is a homolog of RdmB (52% sequence identity) that catalyzes methylation at O-4 of carminomycin (133) yielding daunorubicin (134),^202^ which is a powerful chemotherapeutic agent and close structural analog of β-rhodomycin (126) (Fig. 26A).^203^ Comparison of the crystal structures of RdmB and DnrK demonstrated subtle changes consistent with the divergence in catalytic activities. Thus, the crystal structure of DnrK bound with the methylation product 4-methoxy-ε-rhodomycin T (136, Fig. 26C) and SAH shows alignment between the acceptor hydroxyl group, the transferred methyl and the sulfur of SAH as expected for methyltransferase activity (Fig. 27A).^204^ In contrast, the ternary complex of RdmB bound with SAM and the hydroxylation product 11-deoxy-β-rhodomycin A (DbrA, 131′) revealed an O–C–S angle of 122.9° between the SAM sulfonium and the corresponding hydroxyl oxygen of 131′ suggesting misalignment for efficient nucleophilic displacement (Fig. 27A).^200^

Recent structural comparisons of RdmB and DnrK coupled with extensive mutational studies by Grocholski and co-workers have provided even more insight into this question.^205^ The Michaelis complex of RdmB is solvent inaccessible,^200^ which prevents protonation of the anionic intermediate 129 by water. Closure of the active site is facilitated by a loop that includes Phe300 and blocks the channel connecting the substrate binding site to the external media (Fig. 27C).^205^ In contrast, the corresponding Phe296 in DnrK faces away from the channel leaving the active site of DnrK solvent accessible (Fig. 27D). Notably, DnrK catalyzes the conversion of 127 to 132*in vitro* (Fig. 26B).^205^ Although the timing of methylation at C4–OH is unclear, this observation is consistent with the hypothesis that upon decarboxylation at C15, the resulting anion (*i.e.*, 129) is quenched by water in the DnrK active site.^205^ Moreover, the different orientation of Phe300 in RdmB and Phe296 in DnrK is due to Ser301 in RdmB, which is missing in the primary sequence of DnrK^205^ (Fig. 27B); however, Phe296 in DnrK can be rotated towards the channel thus blocking solvent access by insertion of a serine residue between Phe296 and Thr297, which mimics the Ser301 in RdmB (Fig. 27B).^205^ As expected, this DnrK insertion mutant (DnrK-Ser) did exhibit hydroxylase activity, implying the importance of solvent extrusion to support hydroxylation activity.^205^

**Fig. 28:**
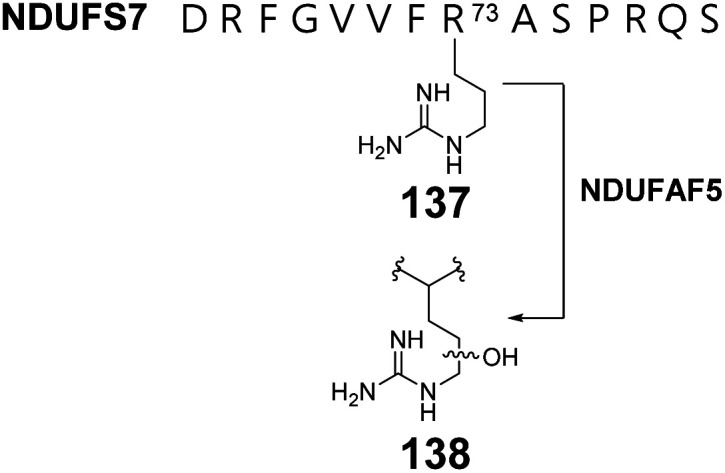
NDUFAF5 catalyzes modification of the substrate protein NDUFS7 at Arg73. The precise site of hydroxylation is unknown.

Human complex I is a respiratory chain protein that drives proton pumping for ATP synthesis by mediating electron transfer between NADH and ubiquinone.^206,207^ NDUFAF5 is an essential protein in the biogenesis of human complex I and is characterized by a conserved SAM-binding motif at the C-terminus. Suppression of NDUFAF5 expression results in reduced hydroxylation of an arginine residue in the protein NDUFS7, which is a subunit in human complex I.^208^ Thus, NDUFAF5 has been hypothesized to be an arginine hydroxylase targeting NDUFS7 (137 → 138) (Fig. 28). The secondary structure of NDUFAF5 is predicted to be similar to that of RdmB in both the substrate and SAM binding regions indicating that NDUFAF5 might adopt an active site structure similar to that of RdmB.^208^ While the *in vivo* activity of NDUFAF5 has been correlated with arginine hydroxylation, the *in vitro* activity and mechanistic details regarding whether the hydroxylase activity is SAM or SAH dependent and how the arginine is activated for oxidation remains unclear.

#### 2.4.5 SAM-dependent decarboxylase

**Fig. 29:**
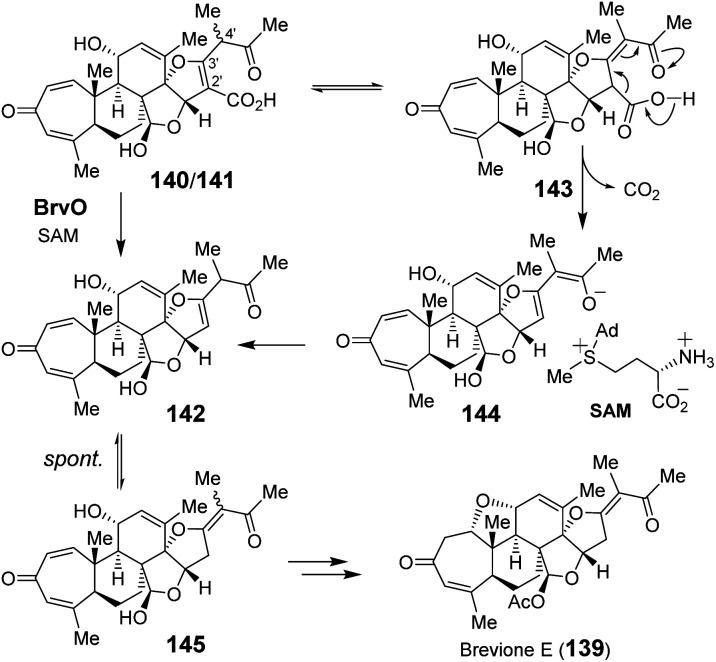
Proposed mechanism for BrvO catalyzed decarboxylation.

Brevione E (139) is a hexacyclic meroditerpenoid demonstrating allelopathic activity.^209^ The biosynthesis of brevione E involves BrvO, which is annotated as a SAM-dependent methyltransferase and proposed to catalyze the decarboxylation of 140/141 to 145 (Fig. 29).^210^ Decarboxylation of 140/141 can be accelerated in the presence of BrvO and SAM. The mechanism is hypothesized to involve initial rearrangement of the 2′,3′-alkene in 140/141 to the 3′,4′-alkene in 143 prior to decarboxylation and generation of the oxyanionic intermediate 144. It is possible that SAM participates by helping to stabilize the negatively charged intermediate (144) *via* electrostatic interactions similar to the role it has been proposed to play during the catalytic cycles of SlnM and RdmB.^196,200^ However, the characterization of BrvO is currently incomplete including whether SAH and SFG can also support BrvO activity. Consequently, further studies will be required to understand the mechanism of this enzyme.

### 2.5 SAM as a nucleophile

#### 2.5.1 Carboxy-SAM

**Fig. 30:**
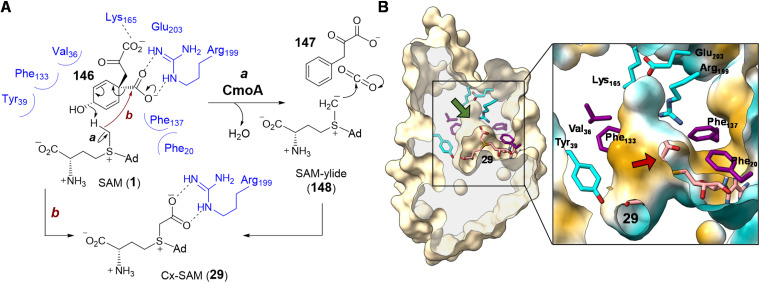
(A) Proposed mechanism for CmoA catalysis. (B) Clip view of Cx-SAM/CmoA complex structure (PDB ID: 4GEK) highlighting the inner cavity for Cx-SAM binding. Computational substrate docking models have suggested that SAM adopts a similar binding configuration as that of the product Cx-SAM. Prephenate (146) is predicted to bind in the hydrophobic pocket adjacent to SAM (green arrow). In the zoomed in region, the surface coloring indicates hydrophobicity (hydrophobic in gold and hydrophilic in cyan). The red arrow indicates the position of the methyl group of SAM, which is surrounded by a hydrophobic environment.

In Section 2.1.3, the formation of 5′-carboxymethyloxyuridine (28) residues at the wobble position of tRNA catalyzed by CmoB was discussed as an example of an alkylation reaction involving carboxy-SAM (Cx-SAM, 29) as the alkyl donor.^86^ However, the biosynthesis of Cx-SAM itself requires carboxylation of the SAM methyl group catalyzed by CmoA with prephenate (146) being the carboxylate donor.^86^ CmoA homologs have also been found in a broad range of organisms and natural product biosynthetic pathways including 3-thiaglutamate (31) in *Pseudomonas* (TglE)^91^ as well as microcin C (34) in *Bacillus* (MccS)^92^ (Fig. 8, Section 2.1.3). The CmoA catalyzed reaction was proposed to involve the nucleophilic addition of a sulfonium methylide (148), generated from α-deprotonation of SAM, to the carbon dioxide liberated upon decarboxylative dehydration of prephenate (Fig. 30A).^86^ Therefore, the CmoA catalyzed reaction differs from other SAM-dependent alkylation reactions in that the methyl substituent on the sulfonium moiety is acting as a nucleophile rather than an electrophile.

The first half of the CmoA catalyzed reaction is hypothesized to proceed analogous to that of prephenate dehydratase, where decarboxylation of prephenate facilitates elimination of a hydroxide ion thereby generating CO_2_ and phenylpyruvate (147).^211^ In the catalytic cycle of prephenate dehydratase, the eliminated hydroxide is believed to abstract a proton from an active site threonine residue thereby completing the reaction.^211^ As the p*K*_a_ of the threonine hydroxyl is close to that measured for the trimethylsulfonium ion,^212^ the same hydroxide is expected to be competent for deprotonating the methyl of SAM. The resulting sulfonium methylide (148) can then add to the eliminated molecule of CO_2_ to yield Cx-SAM (29). Moreover, the X-ray crystal structure of Cx-SAM-bound CmoA from *E. coli* revealed an overall Rossmann fold^86,87^ and a cavity adjacent to the Cx-SAM binding site that was predicted to be the prephenate (146) binding site by molecular docking.^86^ Notably, the region of the active site adjacent to the eliminated prephenate hydroxide is lined primarily with hydrophobic residues leaving the methyl group of SAM the only available proton source nearby (Fig. 30B).^86^

Computational substrate docking has also suggested an arrangement of the prephenate hydroxyl and SAM methyl that disallows a typical methylation reaction.^86^ This is consistent with the observation that CmoA does not catalyze *O*-methylation of prephenate. The docking results instead predicted a 3.4 Å O⋯H–C hydrogen bond between the prephenate hydroxyl group and the SAM methyl, which is more consistent with the proposed deprotonation reaction.^86^ Formation of the putative sulfonium methylide (148) was also supported by the observed exchange of only a single deuterium from CD_3_-labelled SAM with solvent under turnover conditions. Finally, the low dielectric constant of the hydrophobic active site may enhance the basicity of the eliminated hydroxide while increasing the acidity of the SAM methyl.^213–216^ This would address the large difference in p*K*_a_ between water and sulfonium α-carbons measured in polar solvents.^212,217^ Likewise, a hydrophobic substrate binding site has also been proposed in the case of Ecm18 to lower the p*K*_a_ value of a sulfonium intermediate in assisting the formation of a sulfur ylide (80) (Section 2.3.3).^159^ While current evidence points to an intermediary sulfonium ylide (148), the reaction could also occur in a concerted manner avoiding the formation of a discrete ylide intermediate (Fig. 30A, route b).

#### 2.5.2 Queuosine

**Fig. 31:**
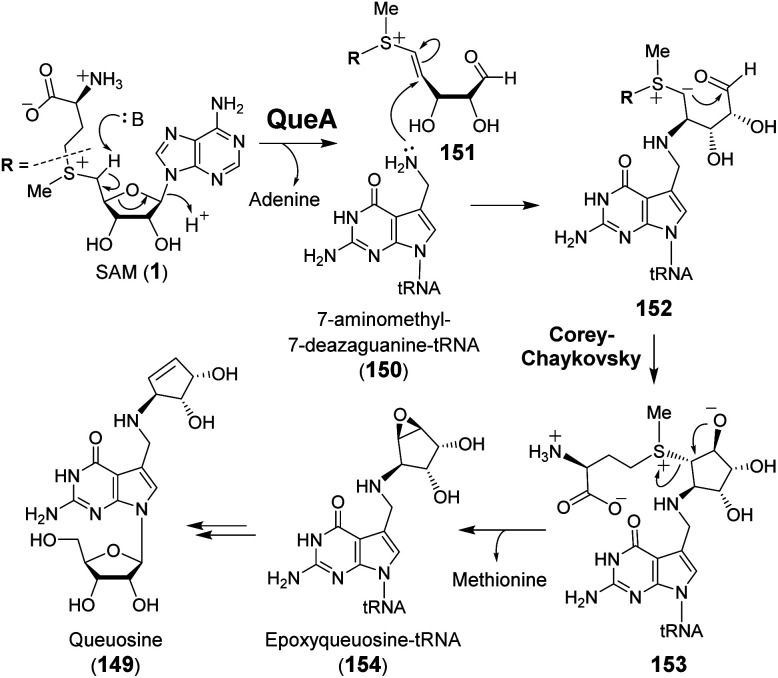
Proposed mechanism of QueA catalyzed ribose transfer reaction.

Apart from CmoA, formation of a sulfonium ylide derived from SAM has also been proposed in the catalytic mechanism of QueA from queuosine (149) biosynthesis.^218,219^ QueA catalyzes transfer and isomerization of the ribose moiety of SAM to a modified guanidine base on tRNA (150). The mechanism was proposed to proceed *via* an initial depurination triggered by deprotonation (1 → 151). Subsequent addition of the substrate amine to the vinyl sulfonium (151) yields sulfur ylide intermediate (152), which can then undergo a Corey–Chaykovsky reaction to give the epoxide product 154 and eliminate methionine (Fig. 31).^218^ The proposed mechanism of QueA is also consistent with kinetic studies showing ordered binding of tRNA followed by SAM to QueA, whereas the order of product release is adenine, methionine and then 154.^220^ However, the only other experimental investigations of QueA catalysis have involved crystallographic studies of the *Thermotoga maritima* and *Bacillus subtilis* enzymes, both structures of which lack bound substrates.^221,222^ Thus, it remains unclear precisely how either tRNA or SAM binds to the enzyme. While the detailed mechanism remains to be explored, QueA represents another potential example of sulfur ylide formation during an enzymatic reaction, which would further expand the chemistry of SAM from an exclusive electrophile to a nucleophile as well.

### 2.6 SAM as an amino donor

**Fig. 32:**
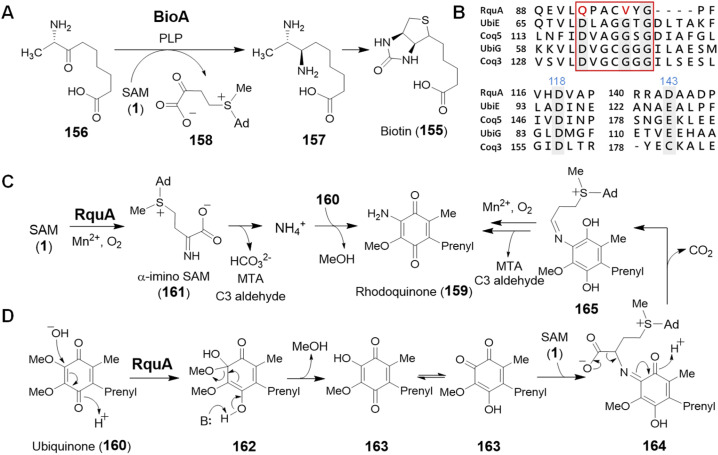
(A) Transamination of SAM catalyzed by BioA. (B) Sequence alignment of RquA with related functional methyltransferases. RquA has two essential aspartate residues (Asp118 and Asp143, numbers in blue) for SAM binding whereas the DXXXGXG (red box) motif typically found in Rossmann fold MTs is missing in RquA. (C and D) Two proposed mechanisms for RquA catalyzed transamination during the biosynthesis of rhodoquinone (159).

SAM is also recognized as an amino donor; however, only two instances of such enzymology have been reported. BioA depends on PLP and catalyzes the oxidative deamination of SAM to its α-keto acid congener while reductively aminating 7-keto-8-aminopelargonic acid (KAPA, 156) to 7,8-diaminopelargonic acid (DAPA, 157) during the biosynthesis of biotin (155) (Fig. 32A).^223,224^ The mechanism of BioA is thus no different from other PLP transaminases,^225^ despite SAM serving as the amine donor rather than a free amino acid. In contrast, the second example appears to be unrelated to PLP chemistry and contributes to the biosynthesis of rhodoquinone in *Rhodospirillum rubrum*.^226^

Rhodoquinone (159) differs structurally from ubiquinone (160) by replacing an *O*-methoxy group with an amine in a reaction that requires the enzyme RquA.^227,228^ RquA was recently characterized as a manganese-dependent enzyme that catalyzes transfer of the α-amino group from SAM to ubiquinone (160) to yield rhodoquinone (159).^229^ While RquA resembles SAM-dependent methyltransferases, only a subset of the conserved residues in the predicted Rossmann fold generally associated with SAM binding are required for activity (Fig. 32B).^227,229^ In addition to a divalent metal ion, RquA catalysis also depends on aerobic conditions as well and is expected to involve a redox component. Indeed, isotope labelling experiments have suggested the oxidative decomposition of SAM to CO_2_, MTA and an aldehyde fragment during the transformation.^229^ Mechanistic hypotheses for this transformation are shown in Fig. 32C and D; however, much work is still left to be done before the chemistry of this unique enzyme is fully understood.

## 3 Enzymes without a typical MT-fold

As discussed in the previous sections, evolutionary diversification of SAM-dependent methyltransferases has led to the emergence of several different types of chemistry. However, there also exist groups of SAM utilizing enzymes that lack the MT-fold making their evolutionary relationship with the SAM-dependent methyltransferases less clear. These enzymes also show a broad range of different activities again emphasizing the mechanistic versatility and intrinsic reactivity of SAM as a biological substrate.

### 3.1 Pyridoxal 5′-phosphate-dependent enzymes

#### 3.1.1 Cyclopropylation

**Fig. 33:**
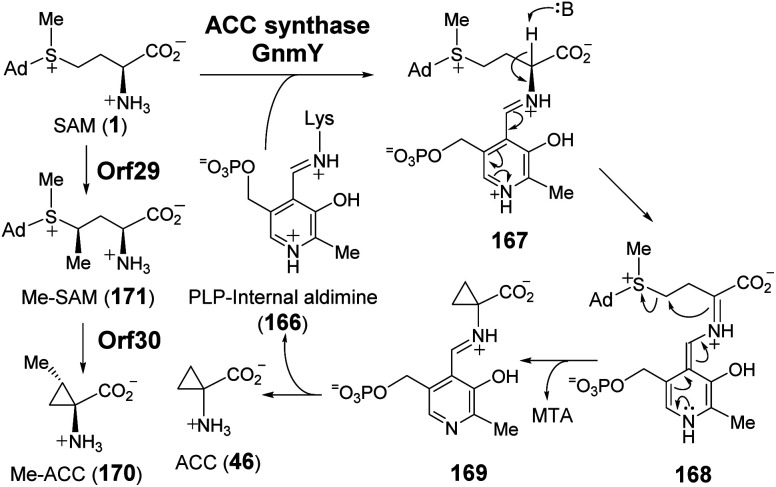
Proposed mechanism for the transformation of SAM to ACC (46) and Me-ACC (170) catalyzed by GnmY and Orf29/Orf30, respectively.

The sulfonium moiety of SAM represents a good leaving group for substitution reactions thereby explaining its prevalence in various biological alkylation reactions. However, this feature of SAM also suggests that it may be susceptible to intramolecular cyclization reactions. Indeed, under alkaline conditions, SAM undergoes nonenzymatic decomposition to yield methylthioadenosine (MTA, 4) and homoserine lactone (44) as one of its main decomposition products.^108,109^ In principle, SAM should also be capable of a favorable 3-*exo*-tet cyclization to form 1-aminocyclopropane-1-carboxylic acid (ACC, 46) again with the elimination of MTA if it were not for the nonacidic C-α proton, which has a p*K*_a_ of approximately 29.^230^ ACC is indeed found as a metabolite from SAM in plants and is the precursor to the important plant hormone ethylene.^231–233^ This difficulty with deprotonation has been overcome in the case of plant ACC synthase by acidifying C1 *via* formation of an external aldimine with PLP (167).^234^ Deprotonation at C1 thus creates a highly conjugated quinonoid (168) and is favorable (Fig. 33). The reaction mechanism of plant ACC synthase has been extensively studied and reviewed;^235^ however, the existence of a bacterial counterpart had been previously unknown despite a number of cyclopropane containing natural products having been isolated from bacteria.^111,112,236–238^ Therefore, it is of interest to know whether similar chemistry is found in bacteria.

**Fig. 34:**
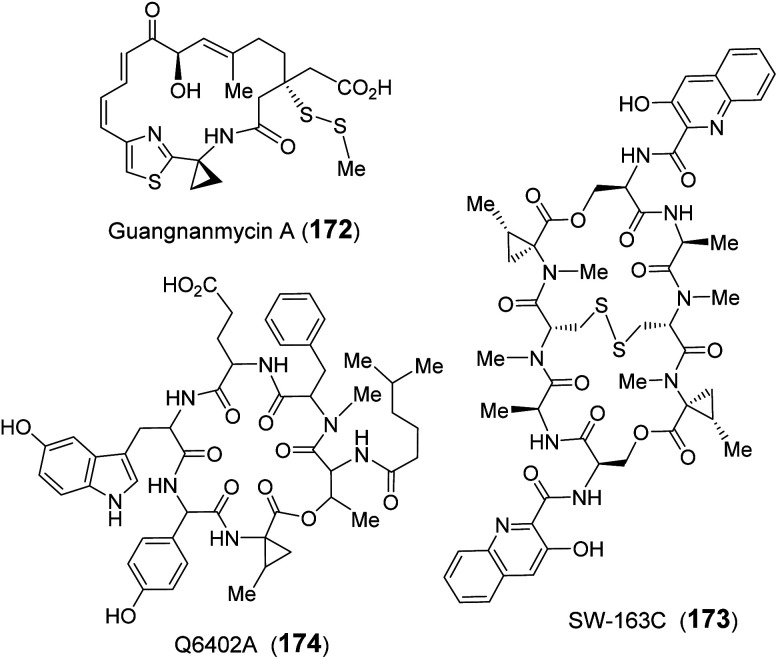
Natural products that contain SAM-derived cyclopropane moieties.

Recently, guangnanmycin (GNM, 172), which is a structural homolog of the antitumor antibiotic leinamycin (LNM), was discovered through genome mining.^239^ Apart from a hybrid peptide–polyketide backbone, GNM also features an unusual ACC moiety (Fig. 34).^239^ Comparative analysis of *lnm*-type biosynthetic gene clusters between guangnanmycin producers and non-producers revealed that the gene product of *gnmY* may catalyze the formation of ACC from SAM. This functional assignment was then validated by *in vivo* inactivation of *gnmY*, which rendered the mutant strain unable to produce guangnanmycin unless exogenous ACC is supplemented.^240^ Sequence analysis revealed that GnmY belongs to the aspartate aminotransferase superfamily, and subsequent *in vitro* characterizations confirmed that GnmY catalyzes the conversion of SAM to ACC in a PLP-dependent manner.^240^ GnmY also exhibits steady-state kinetic parameters comparable to those of plant ACC synthases and utilizes a catalytic lysine residue (Lys251) to form an internal aldimine (166) with PLP during catalysis.^240^ Nevertheless, bioinformatics analyses have suggested that GnmY is phylogenetically distinct from the family of plant ACC synthases despite sharing a similar mechanism of catalysis.^240^

Homologs of GnmY have also been found in the putative biosynthetic gene clusters for two other peptide antibiotics, SW-163C (173) and Q6402A (174),^157,241^ which are each decorated with an unusual 2-methyl-ACC (MeACC, 170) moiety (Fig. 34).^242,243^ In the biosynthetic gene cluster of Q6402A, the gene product of *orf30* shows homology with GnmY suggesting that its activity may involve the formation of MeACC (170).^241^ Initial characterization of purified Orf30 revealed its ability to catalyze the conversion of SAM to ACC.^241^ However, Orf30 exhibits a 1900-fold lower value of *k*_cat_ compared to GnmY such that SAM may not be the native substrate of Orf30.^241^ The B_12_-dependent radical SAM methylase Orf29 encoded by a gene fragment upstream of Orf30 was later demonstrated to catalyze methylation of SAM yielding (4′′*R*)-4′′-methyl-SAM (171).^244^ This SAM derivative (171) can be efficiently cyclized to give MeACC (170) under the action of Orf30 (Fig. 33).^244^ It remains to be established how Orf30 distinguishes between SAM and methylated-SAM.

#### 3.1.2 Elimination

**Fig. 35:**
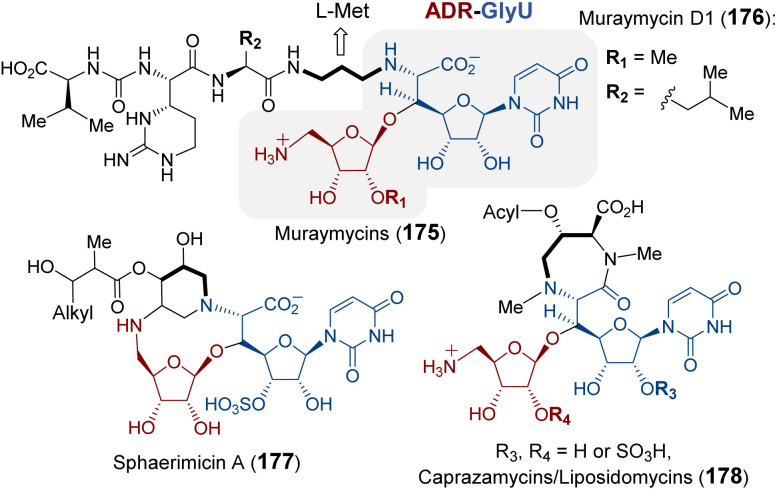
Natural products that contain the ADR-GlyU disaccharide. The bold alkyl chain indicates the putative methionine-derived aminopropyl moiety.

The muraymycins (175), sphaerimicin A (177) and caprazamycins/liposidomycins (178) are peptidyl nucleoside natural products that inhibit bacterial cell wall assembly.^245,246^ These antibiotics are structurally characterized by a unique disaccharide core consisting of 5′′-amino-5′′-deoxyribose (ADR) and 5′-*C*-glycyluridine (GlyU) sugar residues (Fig. 35).^247^ These nucleoside antibiotic families are distinguished, however, by highly diverse appendages at the amino acid end of GlyU. Feeding experiments with a variety of amino acids enriched with stable isotopes revealed that the aminopropyl linker in muraymycin D1 (176) is derived from l-Met (Fig. 35).^248^ Although such a feature could be explained by an ACP transferase catalyzed reaction in the biosynthetic pathway as described in Section 2.1.1 followed by decarboxylation, no ACP transferase or SAM methyltransferase homolog is conserved among the biosynthetic gene clusters for muraymycins or related compounds.^249–252^ These clusters instead encode a PLP-dependent enzyme (*e.g.*, Mur24, Cpz13, LipJ, SphL) with a sequence similar to enzymes annotated as ACC synthases also suggesting SAM as the precursor.^248^

**Fig. 36:**
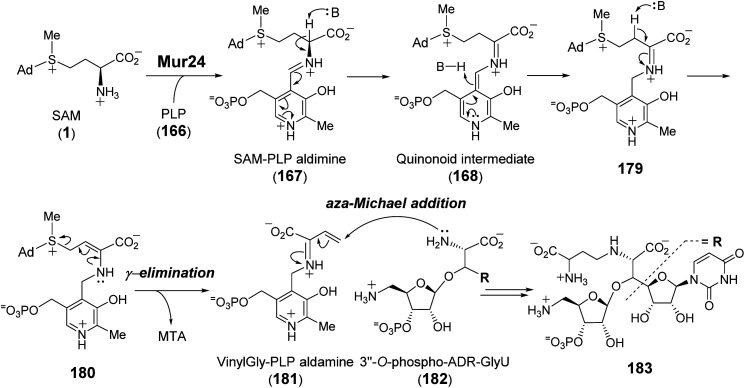
Proposed mechanism for Mur24 catalyzed reaction.

Purification and *in vitro* characterization of Mur24 and its homologs LipJ^250^ and SphL^252^ indicated that all three enzymes do indeed catalyze ACP transfer from SAM to the substrate (182) amine coupled with the elimination of MTA (Fig. 36).^248^ To investigate the role of PLP in this reaction, the conserved lysine residue necessary for internal aldimine formation with PLP was mutated leading to a complete loss of ACP transfer activity.^248^ The catalytic mechanism of Mur24 was further studied using SAM isotopologs, the results of which provided evidence that two protons are washed out in the product (183) including the C-α proton and either the C-β or C-γ proton.^248^ Furthermore, reactions run in D_2_O led to a mixture of both single and double solvent deuterium incorporation into the product 183.^248^ These observations implied sequential and reversible deprotonation at C-α and C-β leading to elimination of MTA following SAM–PLP aldimine (167) formation. The unsaturated iminium intermediate, or vinylglycine–PLP aldamine (181), thereby generated then undergoes aza-Michael addition to yield the *N*-alkylated product 183 (Fig. 36).

**Fig. 37:**
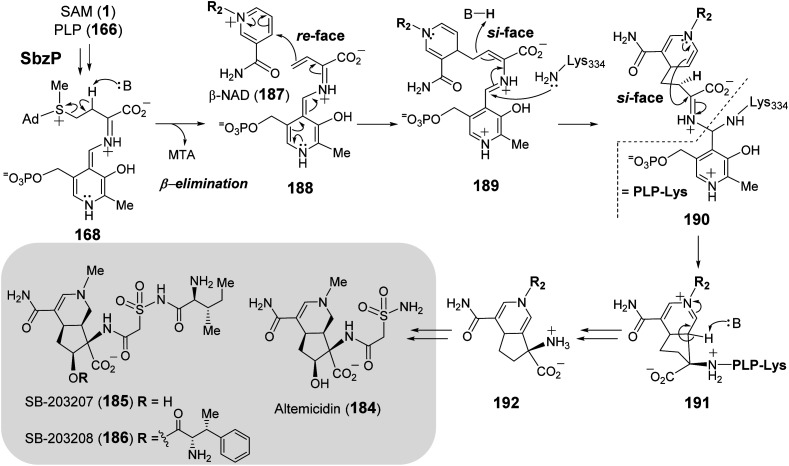
Proposed mechanism of SbzP catalyzed transformation of SAM (1) and β-NAD (187) into 192, which serves as the precursor to 6-azatetrahydroindane containing natural products (184–186).

More recently, the PLP-dependent enzyme SbzP was found to catalyze formation of the 6,5-bicyclic core of 6-azatetrahydroindane containing anticancer natural products (184–186).^253–255^ Feeding experiments established that the biosynthetic origin of the C4 moiety on the five-member ring is likely derived from aspartate and the six-member ring from a nicotinamide containing nucleotide.^256^ Extensive substrate screening corroborated SAM and β-nicotinamide adenine dinucleotide (β-NAD, 187) as the native substrates of SbzP (Fig. 37).^256^ Steady-state kinetic analysis implied a ping-pong Bi–Bi mechanism. Upon incubation of SbzP with SAM, MTA is released with concomitant formation of an enzyme-bound intermediate prior to NAD binding and subsequent formation of the annulation product.^256^

Although the SbzP catalyzed fragmentation of SAM appears similar to that of Mur24 at the point of generating 168, the second half reactions catalyzed by Mur24 and SbzP are distinct. While the unsaturated iminium (181) generated during the Mur24 catalytic cycle is connected to PLP as an aldamine and undergoes alkylation by an incoming nucleophile, during SbzP catalysis, the vinyl glycine iminium 188 instead links to a quinonoid form of PLP and serves to increase the nucleophilicity of C-γ facilitating its addition to the electrophilic nicotinamide ring of NAD (188 → 189) (Fig. 37). Addition of the active site lysine residue covalently links the intermediate 189 with the enzyme as a *gem*-diamine 190 and facilitates rearrangement to the bicyclic structure 192. Spectroscopic analysis of SbzP revealed that addition of SAM results in a unique absorption at 520 nm consistent with the formation of a β,γ-unsaturated quinonoid (188) with an extended π-conjugation as predicted.^256^ An analogous spectroscopic characterization of Mur24 is presently unavailable; however, it would also help provide additional mechanistic insight into how the PLP cofactor participates in reactions with opposite electronic demands.

**Fig. 38:**
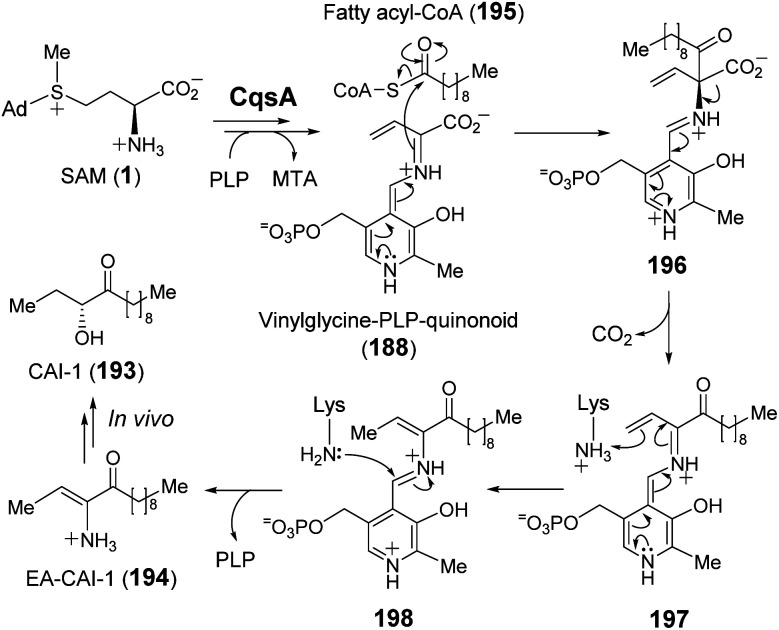
Proposed mechanism of CqsA reaction.

The enzymes described above employ a SAM–PLP quinonoid intermediate (168) that can react *via* several different pathways eliminating MTA to either yield an unsaturated iminium or induce an intramolecular cyclization. In the former case, the iminium is susceptible to both electrophilic and nucleophilic addition. Moreover, electrophilic addition can occur at either C-α or C-γ, with the latter exemplified by SbzP. In contrast, attack at C-α is operative in the enzyme CqsA. Initial study of CqsA (PDB ID: 2WK8) revealed its structural resemblance with other PLP-dependent enzymes^257,258^ and its activity towards different amino acids. However, SAM was later identified as the biosynthetic CqsA substrate to yield EA-CAI-1 (194), which is the precursor to the quorum sensing autoinducer CAI-1 (193) (Fig. 38).^257^ CAI-1-type autoinducers are produced by *Vibrio cholerae*, which is the bacterium that causes the life-threatening disease cholera.^259^ The net transformation of SAM to EA-CAI-1 (194) catalyzed by CqsA involves C–S bond cleavage at C-γ, C–C bond formation at C-α with a fatty acyl-CoA (195) and decarboxylation. The catalytic cycle appears to begin with formation of a PLP–SAM adduct that is converted to a quinonoid intermediate (188) between vinyl glycine and PLP following elimination of MTA as in the case of SbzP. CqsA catalysis then proceeds with nucleophilic addition of the α-carbon to the incoming acyl-CoA substrate (195) before subsequent decarboxylation (196 → 197). The resulting EA-CAI-1 (194) is then converted to CAI-1 (193) by subsequent enzymes *in vivo*.^260^ While the aforementioned transformations exploit the nucleofugality of the sulfonium moiety of SAM, they are not direct S_N_2-type substitution reactions and may also be considered within the context of exotic PLP chemistry, which has been reviewed recently.^261^

### 3.2 Polyketide synthase

#### 3.2.1 Cyclopropylation

**Fig. 39:**
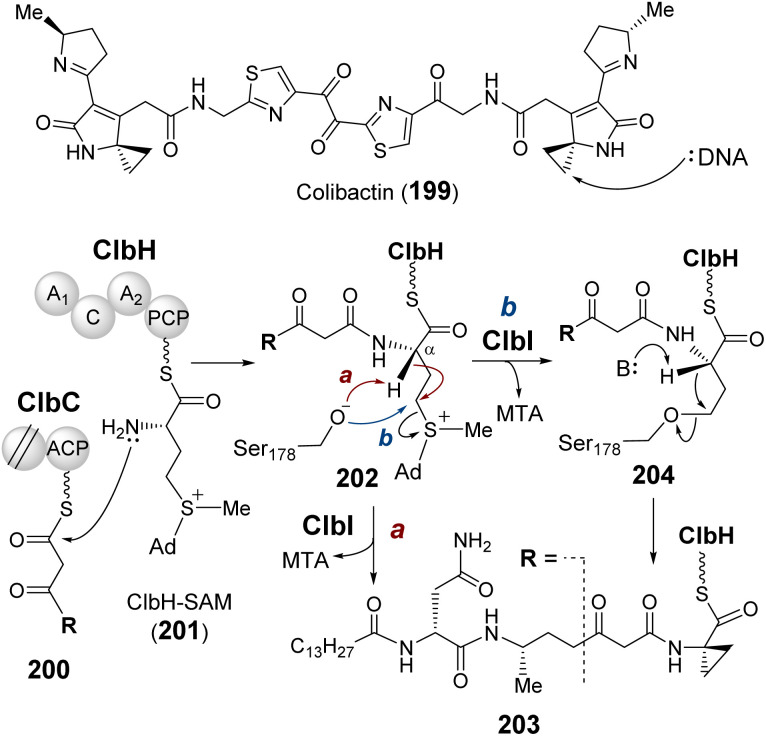
Proposed mechanism for the ClbI catalyzed cyclopropylation during the formation of colibactin biosynthetic intermediate 203.

Colibactin (199) is a genotoxic peptide–polyketide metabolite isolated from *E. coli* featuring a cyclopropane ring, which is believed to be responsible for its DNA alkylation activity.^262–264^ Unlike plant ACC synthases and GnmY, which catalyze the conversion of SAM to free ACC (46),^235,240^ the cyclopropane ring in colibactin is constructed by cyclizing SAM as a component residue during peptide–polyketide chain assembly.^265^ SAM is first loaded onto a peptidyl carrier protein domain in the NRPS protein ClbH followed by a condensation step, which transfers the acyl chain from the upstream PKS (ClbC, 200) to the amino group of ClbH–SAM (201) to yield intermediate 202 (Fig. 39).^265^ ClbI, which is annotated as a PKS enzyme, then catalyzes cyclization of the tethered SAM-acyl chain. Sequence alignment reveals that an otherwise conserved cysteine residue in the ketosynthase (KS) domain of ClbI is replaced by a serine (Ser178), and mutation of this residue to alanine abolishes the activity of ClbI.^265^ It was therefore hypothesized that the serine could act as a general base to deprotonate the α-proton (Fig. 39, mechanism a).^265^ The generated carbanion can then undergo 3-*exo*-tet cyclization and eliminate MTA (202 → 203). Alternatively, serine can also serve as a nucleophile to displace MTA thereby forming an enzyme–substrate adduct 204 (Fig. 39, mechanism b) prior to deprotonation induced cyclization.^265^ As the crystal structure of ClbI or ClbI with SAM-loaded ClbH is currently unavailable, the precise role of the Ser178 and how the non-acidic proton at C-α in 202 is deprotonated is presently uncertain.

## 4 Conclusions

SAM is ranked among the most frequently observed substrates in enzymatic transformations.^18^ The majority of known SAM dependent biological reactions involve methylation; however, regioselective transfer of all three alkyl substituents of the SAM sulfonium have been reported (Sections 2.1 and 2.2) and may represent components of more complex biosynthetic cascades (Section 2.3). In addition to alkyl transfer, several other types of SAM-dependent reactions catalyzed by enzymes have been established including α-deprotonation with the potential for ylide formation (Section 2.5) as well as β-elimination concomitant with elimination of a neutral disulfide (Section 3).

In all cases, these enzyme catalyzed reactions conform to the well-known chemistry of the sulfonium cation, such that the role of the enzyme is to selectively stabilize those transition states representing one mode of reaction over another. Structural investigations of these enzymes have been critical in defining these constraints thereby explaining both the modes of catalysis as well as the observed regiochemistry underlying these various enzyme catalyzed reactions. Moreover, the principles underlying sulfonium enzymology are not limited to SAM-dependent enzymes alone and have also been appreciated among the dimethylsulfoniopropionate (DMSP) lyases found in marine bacteria^266^ as well as BurG during the biosynthesis of malleicyprols.^267,268^

Many aspects of SAM enzymology, however, remain to be explored. In particular, several enzymes as described in Section 2.4 require SAM even though the catalytic cycles of these enzymes do not appear to involve any sort of covalent rearrangement or fragmentation of SAM itself. In these instances, SAM may be required for proper folding and structural integrity of the enzyme,^269^ or it may play a more direct role in catalysis by modifying the structural and electrostatic features of the active site.^270,271^

Many of the SAM-dependent enzymes also exhibit structural features that imply homology with the SAM methyltransferases, whereas others do not, suggesting different evolutionary lineages. Consequently, how these enzymes diversified in some cases from a common evolutionary origin to exhibit the broad range of biochemical activities and unique catalytic roles of SAM seen today is yet another question that remains open to investigation. In any case, SAM has become a focal point of modern enzymology that will likely bring many new discoveries and surprises in the years to come.

## 5 Conflicts of interest

The authors declare no conflicts of interest.

## Supplementary Material
